# Intermittent fasting reduces alpha-synuclein pathology and functional decline in a mouse model of Parkinson’s disease

**DOI:** 10.1038/s41467-025-59249-5

**Published:** 2025-05-14

**Authors:** Éva M. Szegő, Lennart Höfs, Anna Antoniou, Elisabeth Dinter, Nadine Bernhardt, Anja Schneider, Donato A. Di Monte, Björn H. Falkenburger

**Affiliations:** 1 Department of Neurology, TU Dresden, Dresden, Germany; 2 German Center for Neurodegenerative Diseases (DZNE), Bonn, Germany; 3 German Center for Neurodegenerative Diseases (DZNE), Dresden, Germany; 4 Department of Old Age Psychiatry and Cognitive Disorders, University Hospital Bonn, University of Bonn, Bonn, Germany; 5 Department of Pharmaceutical Sciences, University of Vienna, Vienna, Austria; 6 Department of Psychiatry and Psychotherapy, TU Dresden, Dresden, Germany

**Keywords:** Parkinson's disease, Cellular neuroscience, Parkinson's disease

## Abstract

Parkinson’s disease (PD) is a neurodegenerative disorder characterized by dopaminergic neuron degeneration and α-synuclein (aSyn) accumulation. Environmental factors play a significant role in PD progression, highlighting the potential of non-pharmacological interventions. This study investigates the therapeutic effects of intermittent fasting (IF) in an rAAV-aSyn mouse model of PD. IF, initiated four weeks post-induction of aSyn pathology, improved motor function and reduced dopaminergic neuron and axon terminal degeneration. Additionally, IF preserved dopamine levels and synaptic integrity in the striatum. Mechanistically, IF enhanced autophagic activity, promoting phosphorylated-aSyn clearance and reducing its accumulation in insoluble brain fractions. Transcriptome analysis revealed IF-induced modulation of inflammation-related genes and microglial activation. Validation in primary cultures confirmed that autophagy activation and inflammatory modulators (CCL17, IL-36RN) mitigate aSyn pathology. These findings suggest that IF exerts neuroprotective effects, supporting further exploration of IF and IF-mimicking therapies as potential PD treatments.

## Abstract (web summary)

Comparison of intermittent fasting (IF) to a standard diet in a mouse model of Parkinson’s disease demonstrated that IF improved movement, protected dopamine neurons, and enhanced the brain’s clearance process, reducing toxic protein buildup and inflammation.

## Introduction

Parkinson’s disease (PD) is one of the most common neurodegenerative disorders, characterized by the degeneration of dopaminergic neurons of the substantia nigra (SN) and by the presence of intraneuronal, α-synuclein (aSyn) containing inclusions called Lewy bodies^1^. Misfolded and aggregated aSyn species drive neuronal pathology, including mitochondrial dysfunction, transcriptional deregulation, impairment of the auto-lysosomal pathway (ALP) and synaptic dysfunction^2^. Neuroinflammation accelerates disease progression, and can even be sufficient to induce aSyn pathology and degeneration of dopaminergic neurons^3,4^.

A robust body of literature demonstrates that aSyn pathology can be caused by impaired autophagy and is reduced by activating autophagy^5,6^. For instance, blocking autophagy by topical application of bafilomycin A1 potentiates aSyn pathology^7^ whereas transduction of nigral neurons with the lysosomal associated membrane protein 2a reduces aSyn-dependent degeneration of dopaminergic neurons^8^. In order to apply this insight to the treatment of PD, or other neurodegenerative diseases, autophagy needs to be activated continually, or repeatedly, in vivo. In yeast, cultured cells and fruit flies, autophagy can be activated by nutrient deprivation^9^, suggesting that fasting could reduce aSyn pathology in vivo by non-invasively activating autophagy.

Intermittent fasting (IF) is a pattern of time-restricted caloric intake with cycles of nutrient depletion and nutrient availability designed to induce signaling responses—often without weight loss. Effects in the central nervous system require more cycles of IF as compared to the periphery. For instance, only 24 h of fasting is sufficient to induce autophagy in mouse liver^10^, whereas 4 weeks of alternate day fasting are required to induce autophagy in the spinal cord^11^. Importantly, IF-induced cellular responses are not restricted to autophagy. For example, IF reduces both basal^12^ and LPS-induced neuroinflammation in the hippocampus^13^, increases BDNF levels in the brain^14–16^, and reduces degeneration of dopaminergic neurons induced by the mitochondrial toxin MPTP or mitochondrial dysfunctions in a mouse model of AD^17,18^.

In this work, we tested the hypothesis that IF reduces aSyn pathology and toxicity in a viral vector-based mouse model of PD. The substantia nigra (SN) was transduced with recombinant adeno-associated viral vectors (rAAV2/7) encoding human αSyn with the disease-related A53T mutation or green fluorescent protein (GFP) as control. Four weeks after transduction, animals were subjected to a 4-week course of alternate day IF diet. The integrity of the nigrostriatal dopaminergic neurons, aSyn pathology, glial response and motor performance were analyzed. Autophagic vesicle pools in the substantia nigra were analyzed in autophagy reporter mice. Transcriptomic analysis was used to study the modulation of signaling pathways in the midbrain. In order to explore potential mechanisms, we exposed primary mouse cultures expressing aSyn to small molecule modulators of selected signaling pathways identified in the transcriptomic analysis and analyzed the inflammatory response, aSyn pathology and toxicity.

## Results

### Intermittent fasting reduces aSyn-induced degeneration of dopaminergic neurons

**Fig. 1:**
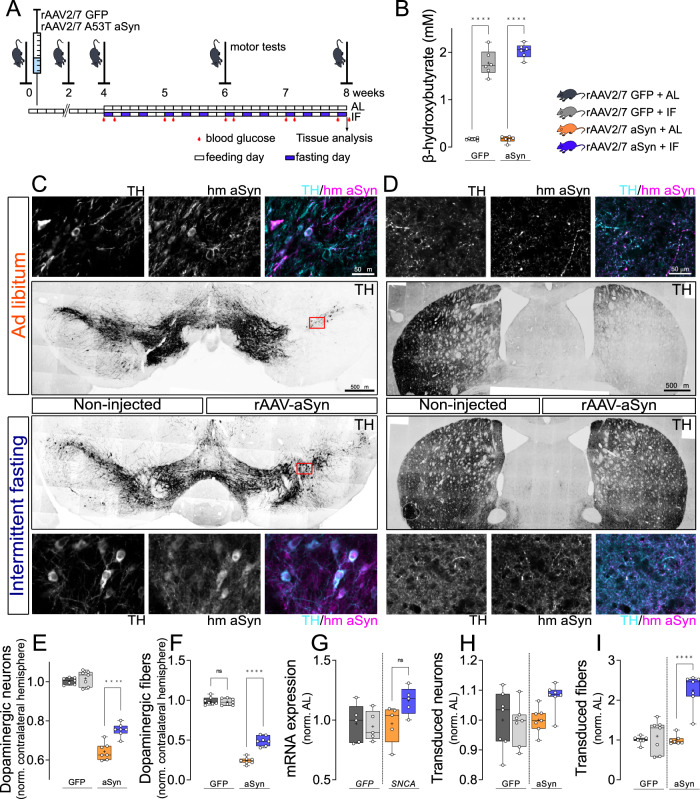
Intermittent fasting reduces aSyn-induced degeneration of dopaminergic neurons. **A** Experimental scheme. **B** Serum levels of BHB (mM). *n* = 6, two-way ANOVA, Tukey post-hoc test. ****: p < 0.0001. **C** Representative images of midbrain sections. Upper images: rAAV-aSyn–AL, lower images: rAAV-aSyn–IF. Overview: TH staining (gray). Scale bar: 500 µm. Insets: TH and human aSyn (hm aSyn) staining (gray), or both (TH: cyan, hm aSyn: magenta) of the indicated areas. Scale bar: 50 µm. **D** Representative images of striatal sections. Upper images: rAAV-aSyn–AL, lower images: rAAV-aSyn–IF. Overview: TH staining (gray) of both hemispheres. Scale bar: 500 µm. Insets: TH and human aSyn (hm aSyn) staining (gray), or both as overlay (TH: cyan, hm aSyn: magenta). Scale bar: 50 µm. **E** Normalized number of TH-positive neurons in the SN. Numbers were normalized to the non-injected hemisphere of the same animal. Box and whisker plots, middle line: median, +: mean, circles: individual animals; *n* = 7, two-way ANOVA, Tukey post-hoc test, p < 0.0001. TH neuron numbers are on Supplementary Fig. 1D. **F** Density of TH-positive fibers in the STR, normalized to the (i) contralateral hemisphere and (ii) to the rAAV-GFP–AL group. Box and whisker plots (see “methods”), middle line: median, +: mean, circles: individual animals; *n* = 7, two-way ANOVA, Tukey post-hoc test, p < 0,0001). Fiber densities are on Supplementary Fig. 1E. **G** Expression of GFP and hm*SNCA* in the SN. Box and whisker plots, middle line: median, +: mean, circles: individual animals; *n* = 5, two-way ANOVA, Tukey post-hoc test. **H** Normalized number of GFP- or human aSyn-positive neurons in the SN. Numbers were normalized to the (i) non-injected hemisphere of the same animal and (ii) AL. Box and whisker plots, middle line: median, +: mean, circles: individual animals; *n* = 7, two-way ANOVA, Tukey post-hoc test, p < 0.0001. **I** Density of GFP- or hmaSyn-positive fibers in the STR, normalized to the (i) contralateral hemisphere and (ii) to rAAV-GFP–AL. Box and whisker plots, middle line: median, +: mean, circles: individual animals; *n* = 7, two-way ANOVA, Tukey post-hoc test, p < 0.0001). Box and whiskers plots: box: 25th to 75th percentiles, whiskers: from the smallest to the largest value. Source data are provided as a Source Data file.

α-Synuclein pathology and neurodegeneration were induced in mice by injecting serotype 2/7 rAAV encoding human aSyn with the disease-linked A53T mutation (rAAV-aSyn), into the substantia nigra (SN, Fig. 1A). rAAV encoding GFP were used as controls. We observed dopaminergic neurons, characterized by tyrosine hydroxylase (TH) staining, that were also positive for human aSyn (Fig. 1C) or GFP (Supplementary Fig. S1H)—as expected. At 8 weeks after surgery, the number of dopaminergic neurons in the injected SN was 35% lower in mice transduced with rAAV-aSyn than in mice transduced with rAAV-GFP (Fig. 1E). In the striatum, we observed fiber- and bead-like structures positive for TH and human aSyn (Fig. 1C), or TH and GFP (Supplementary Fig. S1J). This is consistent with the transduction of dopaminergic neurons in the SN and consecutive expression of human aSyn or GFP in dopaminergic axon terminals in the striatum. The density of dopaminergic axon terminals in the striatum was 75% lower in mice transduced with rAAV-aSyn than in mice transduced with rAAV-GFP (Fig. 1F). The more pronounced degeneration of dopaminergic axon terminals in the striatum as compared to dopaminergic somata in the SN is commonly observed in animal models of PD^19^.

The alternate day intermittent fasting (IF) diet was applied in this mouse model, starting 4 weeks after rAAV injection (Fig. 1A). At this time point, dopaminergic neurons in the SN were already reduced by 20% (Supplementary Fig. S1F), consistent with previous findings^20^. During the subsequent 4 weeks of IF diet, mice were housed in cages without food pellets on Mondays, Wednesdays and Fridays, and in cages with pellets ad libitum on all other days. Control mice were also transferred between cages, but all cages contained food pellets (ad lib. feeding, AL). In the IF group, the weight of mice declined by about 6% during each day of fasting, and recovered during the following day (Supplementary Fig. S1B). Therefore, the overall weight was not different between IF mice and AL mice at the end of the 4 weeks course (Supplementary Fig. S1A). Similarly, blood glucose declined by 45% after fasting in the IF group, but recovered on the subsequent day (Supplementary Fig. 1C). These findings about weight and glucose are consistent with previous data^21–23^.

During fasting, ketone bodies like beta-hydroxybutyric acid (BHB) are synthesized in the liver and in astrocytes^24,25^. BHB represents not only an essential energy carrier, but also regulates signaling pathways, metabolism and gene transcription^26–28^. BHB levels in the serum of mice with IF diet were elevated as compared to mice with AL diet (Fig. 1B), which is consistent with previous findings by others^29,30^ and confirms the efficacy of the IF diet.

In mice transduced with rAAV-aSyn and subjected to the IF diet (aSyn-IF mice), the degeneration of dopaminergic neurons in the SN was less pronounced (33% less lesion) than in mice transduced with rAAV-aSyn on AL diet (aSyn-AL mice, Fig. 1D). Similarly, the degeneration of dopaminergic axon terminals in the striatum was less pronounced in aSyn-IF mice than in aSyn-AL mice (30% less lesion, Fig. 1F). In mice transduced with rAAV-GFP, there was no difference between IF and AL diet (GFP-IF and GFP AL mice, Fig. 1E, F).

This reduction in dopaminergic neuron degeneration by IF did not result from altered rAAV transduction, because the expression of the human *SNCA* mRNA, as quantified by qPCR from the SN, was not different after 8 weeks in aSyn-IF mice compared to aSyn-AL mice (Fig. 1G), and the density of fibers positive for human aSyn in the striatum was higher after IF diet as compared to AL diet (Fig. 1I). For the numbers of transduced neurons in the SN, we observed a similar pattern without significant difference between IF and AL (Fig. 1H). These observations can rather be explained by the blunted degeneration of dopaminergic neurons in the IF group (Fig. 1E, F). The expression of the *GFP* mRNA was not different between AL and IF mice (Fig. 1G), and there was no difference in the density of GFP-positive fibers between AL and IF (Fig. 1I).

Taken together, our data show that 4 weeks of IF reduced degeneration of dopaminergic neurons and their striatal axon terminals transduced by rAAV-aSyn.

### Reduced neurodegeneration with intermittent fasting is functionally relevant

**Fig. 2:**
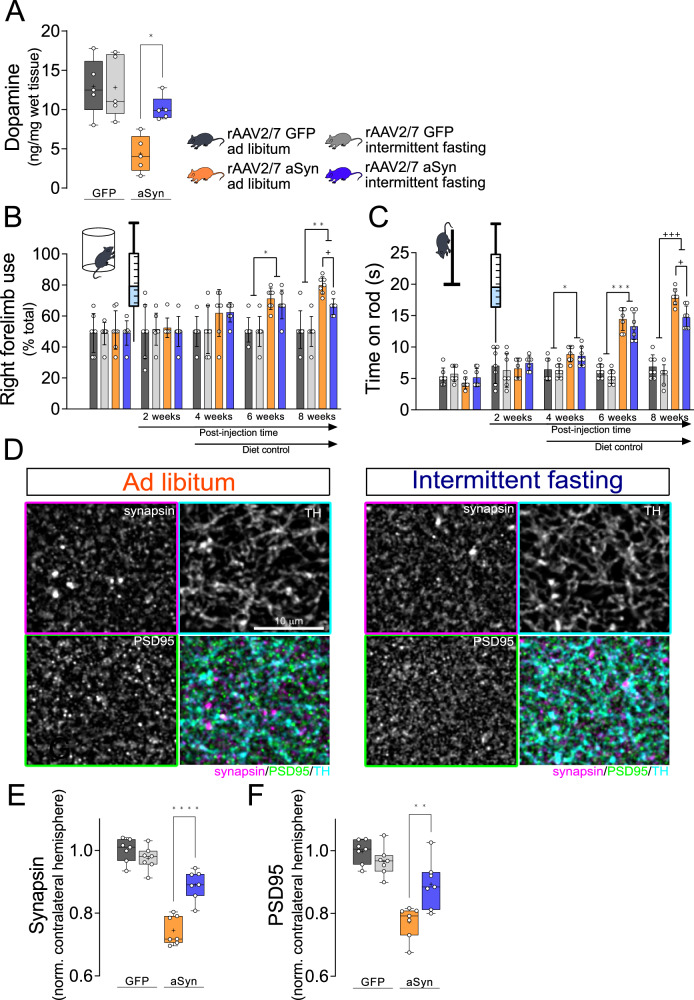
Intermittent fasting improves aSyn-induced motor impairment and reduces striatal synapse loss. **A** Dopamine content (ng/mg wet tissue) in striatal lysates. Graph shows box and whisker plots, middle line: median, +: mean, circles: individual animals; *n* = 5, two-way ANOVA, Tukey post-hoc test, *p* = 0.023. **B** Quantification of the unilateral motor deficits (asymmetry in forelimb use) using the cylinder test. Mice were tested before and 2, 4, 6, and 8 weeks after the vector injection. Mean ± SD, circles: individual animals; *n* = 7, repeated measures ANOVA, Tukey post-hoc test; *: *p* = 0.012; **: *p* = 0.0022; +: *p* = 0,0242. Graph showing the time until the first 25 paw contacts is on Supplementary Fig. 3A. **C** Quantification of the motor coordination using the rod test. Mice were tested before and 2, 4, 6, and 8 weeks after the vector injection. Mean ± SD, circles: individual animals; *n* = 7, repeated measures ANOVA, Tukey post-hoc test; *: *p* = 0.0232; ***: *p* = 0.0008; +++: *p* = 0.0006; +: *p* = 0.018. **D** Representative images of striatal sections from rAAV-aSyn-injected mice stained for the presynaptic marker synapsin (magenta in overlay images), for the postsynaptic marker PSD95 (green) and TH (cyan). Scale bar: 10 μm. **E** Quantification of area fraction positive for the presynaptic marker synapsin in the STR. Numbers were normalized (i) to the contralateral hemisphere of the same animal and to the (ii) rAAV-GFP-AL group. Graph shows box and whisker plots, middle line: median, +: mean, circles: individual animals; *n* = 7, two-way ANOVA, Tukey post-hoc test, p < 0.0001. Graph showing area fractions from both hemispheres are on Supplementary Fig. 3B. **F** Quantification of the area fraction positive for the postsynaptic marker PSD95 in the STR. Numbers were normalized (i) to the contralateral hemisphere of the same animal and to the (ii) rAAV-GFP—AL group. Graph shows box and whisker plots, middle line: median, +: mean, circles: individual animals; *n* = 7, two-way ANOVA, Tukey post-hoc test, p < 0.0077. Graph showing area fractions from both hemispheres are on Figure supplement 3C. Box and whiskers plots: box: 25th to 75th percentiles, whiskers: from the smallest to the largest value. Source data are provided as a Source Data file.

In order to validate the effect of IF diet on the functional integrity of dopaminergic axon terminals, we carried out a second set of experiments (experiment 2) in the same way as described in Fig. 1A except that rAAV was injected into both hemispheres to reduce the number of animals needed. The concentration of dopamine was measured in striatum lysates (Fig. 2A). In animals with AL diet, striatal dopamine concentration was 66% lower with rAAV-aSyn transduction than with rAAV-GFP transduction. With IF diet, this decline was smaller, dropping only by 21 % as compared to GFP-IF mice (Fig. 2A, 68% of the aSyn-induced dopamine loss was rescued by IF diet). IF had no effect on striatal dopamine in animals transduced with rAAV-GFP.

The functional consequences of unilateral dopamine depletion in the striatum include asymmetry in forepaw use^31–33^. To examine whether IF was also effective in reducing the aSyn-induced motor phenotype, we quantified forepaw use in the cylinder test at different time points during the experimental procedure (Fig. 1A). At baseline and 2 weeks after surgery, both forepaws were equally used (Fig. 2B), and the time to complete 25 forepaw contacts with the cylinder wall remained unchanged (Supplementary Fig. 2A). Starting at 6 weeks after surgery, the relative use of the right forepaw increased in all rAAV-aSyn-injected mice (Fig. 2B). Eight weeks after rAAV-aSyn injection, aSyn-AL mice used their right forepaw in 80% of cases. In addition, the time to complete 25 contacts nearly doubled (Supplementary Fig. S2A). This aSyn-dependent motor phenotype was blunted in aSyn-IF mice at 8 weeks after surgery, i.e., after 4 weeks of IF diet (Fig. 2B; Supplementary Fig. S2A).

In addition, we measured general motor performance (coordination and balance) using the vertical rod test—with similar findings as for the cylinder test (Fig. 2C): Motor performance started declining at 4 weeks after rAAV-aSyn transduction and was blunted with IF diet at 8 weeks after surgery, i.e., after 4 weeks of IF (Fig. 2C). In both motor tests, IF did not affect motor performance in animals transduced with rAAV-GFP.

Reduced dopaminergic innervation of the striatum entails the reduction of synaptic connections^34^ and also motor performance can affect striatal synapses^35^. Therefore, we next assessed synaptic density in the striatum by staining presynaptic structures for synapsin and postsynaptic structures for PSD95 (Fig. 2D–F, Supplementary Fig. 2B, C). In aSyn-AL animals, the density of synapsin-positive puncta was 25% lower than GFP-AL mice (Fig. 2D, E). This aSyn-induced decline was blunted to 11% by IF (comparing aSyn-IF and GFP-IF mice). Similarly, the density of PSD95-positive puncta was 22% lower in aSyn-AL mice than in GFP-AL mice (Fig. 2D, F). With IF, this aSyn-induced decline was blunted to 10 % (not significant difference between GFP-IF and aSyn-IF mice; Fig. 2F). Interestingly, IF induced a small, but statistically significant increase in the density of synapsin-puncta and PSD95 puncta of both hemispheres, in mice transduced with rAAV-GFP (Supplementary Fig. 2B, C).

As additional markers for synaptic integrity, we measured expression of glial-derived neurotrophic factor (GDNF) and brain-derived neurotrophic factor (BDNF), which are modulated by fasting^36,37^. The mRNA of *Gdnf*, as measured by qPCR from the striatum lysates, was not significantly different in mice transduced by rAAV-aSyn as compared to rAAV-GFP, and was not affected by diet (Supplementary Fig. 2D). In contrast, mRNA of *Bdnf* was higher in mice on IF diet than in mice on AL diet (Supplementary Fig. 2D). This effect was observed both in mice transduced by rAAV-GFP and in mice transduced by rAAV-aSyn, but the induction was more pronounced in mice transduced by rAAV-aSyn.

In summary, IF blunted the aSyn-induced reduction in striatal dopamine, the aSyn-dependent motor impairment, and the reduction in synaptic structures. These findings indicate that the protective effects of IF on aSyn-induced neurodegeneration are functionally relevant.

### Increased autophagosomal engulfment of toxic aSyn species and autophagosome maturation in dopaminergic neurons of IF mice

**Fig. 3:**
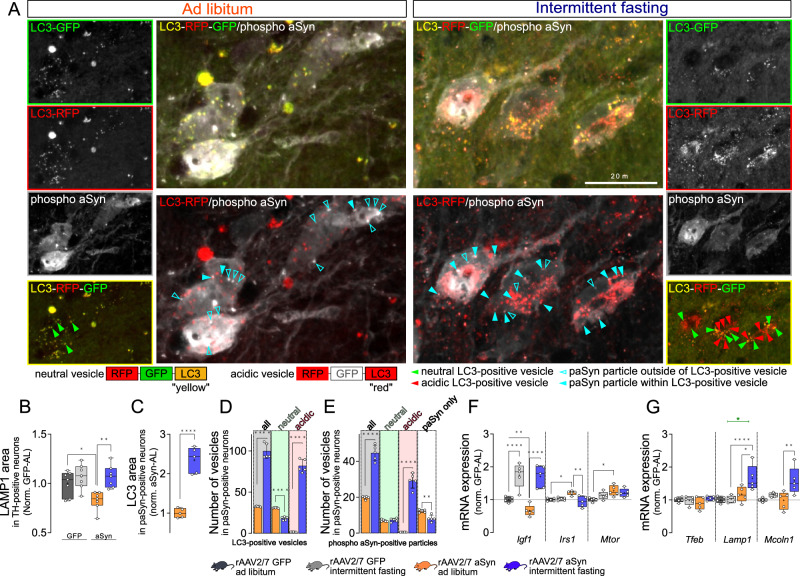
Intermittent fasting induces autophagy, autophagosome maturation and increased colocalization of LC3 and phospho-aSyn in the SN. **A** Representative images of SN sections stained for phospho-aSyn (white). tfl-LC3 construct is visualized in green (EGPF) and red (RFP) (top images), or only in red (bottom). Small images (left and right): individual channels. Green arrowheads: neutral LC3-positive vesicles; red arrowheads: acidic LC3-positive vesicles; open blue arrowheads: free phospho-aSyn particles outside of autophagosomes; blue arrowheads: phospho-aSyn-positive particles within LC3-positive vesicles. Scale bar: 20 μm. Lower magnification images: Supplementary Fig. 5C. **B** Area fraction positive for LAMP1 within TH-positive neurons in the SN. Numbers were normalized to rAAV-GFP–AL. Box and whisker plots, middle line: median, +: mean, circles: individual animals; *n* = 7, two-way ANOVA, Tukey post-hoc test, *: *p* = 0.042; **: *p* = 0.0069. **C** Area fraction positive for LC3 signal (red channel) within phospho-aSyn-positive neurons. Numbers were normalized to rAAV-aSyn–AL. Box and whisker plots, middle line: median, +: mean, circles: individual animals; *n* = 5, two-sided t-test, p < 0.0001. Area fractions of LC3 signal in the SN are on Figure supplement 5A. **D** Average number of total, neutral and acidic LC3-positive vesicles within phospho-aSyn-positive neurons. Box and whisker plots, middle line: median, +: mean, circles: individual animals; *n* = 5, two-sided t-test, ****: p < 0.0001. **E** Average number of phospho-aSyn-positive particles within neutral or acidic LC3-positive vesicles, or outside. Box and whisker plots, middle line: median, +: mean, circles: individual animals; *n* = 5, two-sided t-test, ****: p < 0.0001; **: *p* = 0.0017. Average vesicle numbers in different positions (shrink analysis) are on Supplementary Fig. 5C, D). **F** Expression of autophagy-related genes in the SN. Box and whisker plots, middle line: median, +: mean, circles: individual animals; *n* = 5, two-way ANOVA, Tukey post-hoc test (*Igf1*: ****: p < 0.0001; **: *p* = 0.0073) (*Irs1*: *: *p* = 0.032; **: *p* = 0.0065) (*Mtor*: *: *p* = 0.039). **G** Expression of lysosome-related genes in the SN. Box and whisker plots, middle line: median, +: mean, circles: individual animals; *n* = 5, two-way ANOVA, Tukey post-hoc test (*Lamp1*: ****: p < 0.0001; *: *p* = 0.029; interaction: *: *p* = 0.013) (*Mcoln1*: **: *p* = 0.0017). Green line and * represent interaction between the treatments (vector and diet). Box and whiskers plots: box: 25th to 75th percentiles, whiskers: from the smallest to the largest value. Source data are provided as a Source Data file.

In order to confirm that IF diet affects autophagy in the brain, we used transgenic mice expressing the autophagosome marker LC3 coupled to the RFP-GFP “tandem-fluorescence” tag (tfl-LC3)^38,39^. This construct allows the visualization of autophagosomes, which appear yellow in images of merged red and green channels. In addition, tfl-LC3 provides information about the maturation of autophagosomes, since RFP is more resistant to acidification (pKa 4,5), whereas GFP is quenched at higher pH (pKa 5,9). Therefore, in acidic vesicles, representing autolysosomes and amphisomes, tfl-LC3 fluorescence appears red on merged images (Fig. 3A). tfl-LC3 transgenic mice were treated as above (Fig. 1A) with the only exception that control mice were injected with PBS instead of rAAV-GFP to avoid interference with the tfl-LC3 transgene. Similar to the previous experiments, IF or AL diet was started 4 weeks after surgery and continued for 4 weeks.

The overall area of LC3-positive structures in the SN was higher with IF diet than with AL (Fig. 3A, Supplementary Fig. 3A). Similarly, the area positive for the lysosomal marker LAMP1 was higher in mice with IF diet than with AL diet (Fig. 3B, Supplementary Fig. 3B). Both results are consistent with findings in spinal cord^11^ and confirm that IF affects the autolysosomal system in the dopaminergic neurons of the SN.

In order to analyze the effects on autolysosomal vesicles in the SN in more detail, we quantified the density of LC3-positive structures within neurons, positive for aSyn phosphorylated at position S129 (phospho-aSyn), a characteristic posttranslational modification for pathological aSyn species^40,41^ (Fig. 3A, C). The LC3-positive area fraction was >2-fold higher in animals with IF than in animals with AL diet (Fig. 3C). Accordingly, the number of LC3-positive vesicles in phospho-aSyn positive neurons was higher with IF than with AL (Fig. 3D). When we discriminated between vesicles with neutral and acidic pH, we observed a decrease in the number of neutral vesicles and an increase in the number of acidic vesicles with IF (Fig. 3D), suggesting improved autophagosome maturation. This finding is consistent with the higher fraction of LAMP1-positive structures (Fig. 3B). We also analyzed the subcellular distribution of LC3-positive vesicles in phospho-aSyn-positive neurons of the SN (Supplementary Fig. 3C, D), and acidic, phospho-aSyn containing LC3 particles localized more centrally in IF mice (Supplementary Fig. 3D). Similarly, a more central subcellular location of lysosomes was observed in cultured cells under starvation conditions^42^.

In addition, we quantified the colocalization of phospho-aSyn-positive structures with LC3-positive vesicles in the SN (Fig. 3E). The overall number of phospho-aSyn-positive particles was >2-fold higher in mice with IF than in mice with AL. However, when we discriminated between three different types of phospho-aSyn-positive particles, the number of phospho-aSyn particles colocalizing with a mature autophagosome marker (acidic and LC3-positive, quenched green fluorescence) was higher in mice with IF. Conversely, the number of phospho-aSyn particles without colocalization to LC3 was lower with IF (Fig. 3E). These findings suggest that “free”, cytoplasmic phospho-aSyn aggregates were reduced by IF whereas the presence of phospho-aSyn in mature autophagosomes was increased.

In order to test whether a transcriptional response to IF diet underlies these effects, we measured the expression of *Mtor*, the master negative regulator of autophagy, and its upstream signaling partners insulin-like growth factor 1 (*Igf1*) and insulin receptor substrate 1 (*Irs1*). Consistent with the known inhibition of autophagy by aSyn^43^, *Mtor* expression was increased in the SN of aSyn-AL mice as compared to GFP-AL control (Fig. 3F). Expression of *Igf1* was reduced in the SN of aSyn-AL mice, and expression of *Irs1* was increased (Fig. 3F). IF diet increased *Igf1* expression, both in mice transduced with rAAV-aSyn and in controls (Fig. 3F). IF normalized the aSyn-induced expression of *Irs1* in mice transduced with rAAV-aSyn and did not significantly affect expression of *Mtor* (Fig. 3F). *Igf1* protects dopaminergic neurons against aSyn toxicity^44^. Increased *Igf1* expression could therefore potentially mediate the neuroprotective effects of IF (Fig. 1).

In order to explain the increased abundance of lysosomal compartments (Fig. 3B) and the higher fraction of mature autophagosomes in phospho-aSyn-positive neurons with IF (Fig. 3D, E), we measured expression of *Tfeb*, a major regulator of lysosomal biogenesis. *Tfeb* expression was not affected by aSyn or IF (Fig. 3G). Expression of the lysosome-related genes *Lamp1* and *Mcoln1*, however, was higher in aSyn-IF mice than in all other groups (Fig. 3G).

Taken together, we observed an increased abundance of autolysosomal vesicles and improved autolysosomal fusion in the SN of aSyn-transduced mice with IF.

### Intermittent fasting reduces accumulation of toxic aSyn species

**Fig. 4:**
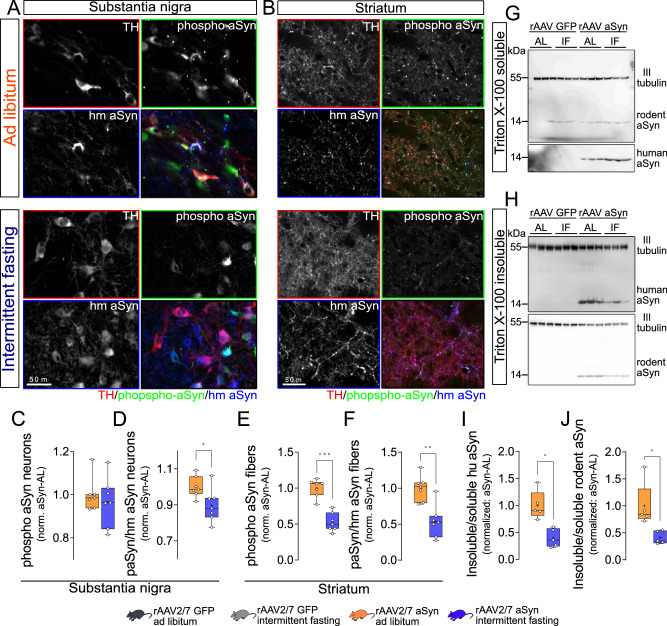
Intermittent fasting reduces alpha-synuclein pathology. **A** Representative, high magnification images of the SN from animals, stained for TH (red on overlay images), phospho-aSyn (green) and human aSyn (blue). Scale bar: 50 µm. **B** Representative, high magnification images of the STR from animals, stained for TH (red on overlay images), phospho-aSyn (green) and human aSyn (blue). Scale bar: 50 µm. **C** Normalized number of phopspho-aSyn-positive neurons in the SN. Numbers were normalized to the rAAV-aSyn–AL group. Box and whisker plots, middle line: median, +: mean, circles: individual animals; *n* = 7, two-sided t-test. **D** Ratio of phospho-aSyn-positive neurons within human aSyn-positive neurons in the SN. Numbers were normalized to the rAAV-aSyn–AL group. Box and whisker plots, middle line: median, +: mean, circles: individual animals; *n* = 7, two-sided t-test, *p* = 0.0282. **E** Normalized density of phospho-aSyn-positive fibers in the STR. Numbers were normalized to the rAAV-aSyn–AL group. Box and whisker plots, median: middle line; mean: +, circles: individual animals; *n* = 7, two-sided t-test, *p* = 0.0006. **F** Ratio of phospho-aSyn-positive fibers within human aSyn-positive fibers in the STR. Numbers were normalized to the rAAV-aSyn–AL group. Box and whisker plots, middle line: median, +: mean, circles: individual animals; *n* = 7, two-sided t-test, *p* = 0.0076. **G** Representative immunoblots of the Triton X-100 soluble fraction showing rodent aSyn and βIII tubulin (upper panel), and human aSyn (lower panel) signal. **H** Representative immunoblots of the Triton X-100 insoluble fraction showing human aSyn and βIII tubulin (upper panel), and rodent aSyn (lower panel) signal. **I** Ratio of the human αSyn signal in the Triton X-100 insoluble and Triton X-100 soluble fractions. Numbers were normalized to the rAAV-aSyn–AL group. Box and whisker plots, middle line: median, +: mean, circles: individual animals; *n* = 5, two-sided t-test, *p* = 0.0359. **J** Ratio of the endogenous αSyn signal in the Triton X-100 insoluble and Triton X-100 soluble fractions. Numbers were normalized to the rAAV-aSyn–AL group. Box and whisker plots, middle line: median, +: mean, circles: individual animals; *n* = 5, two-sided t-test, *p* = 0.0148. Box and whiskers plots: box: 25th to 75th percentiles, whiskers: from the smallest to the largest value. Source data are provided as a Source Data file.

In order to determine whether the effects of IF on autophagy translate into alteration of synuclein pathology, we assessed aSyn pathology as assessed by staining against phospho-aSyn in the SN. Numerous neurons positive for human aSyn and phospho-aSyn were observed in the SN of the hemisphere transduced by rAAV-aSyn. Many, but not all, were also positive for TH (Fig. 4A). The number of phospho-aSyn-positive neurons was similar in aSyn-AL and aSyn-IF mice (Fig. 4C). More TH-positive neurons in the SN degenerated in aSyn-AL than in aSyn-IF mice (Fig. 1D), and we assumed that the reduced formation of the pathological phospho-aSyn species in aSyn-IF mice might be responsible for it. Therefore, we next determined the ratio of phospho-aSyn-positive neurons among the transduced, human aSyn-positive neurons. In this comparison, fewer of the transduced neurons showed aSyn pathology as reported by phospho-aSyn staining in aSyn-AL than in aSyn-IF mice (Fig. 4D).

We further determined the extent of phospho-aSyn pathology in the striatum. Indeed, we observed a substantial amount of human-aSyn positive fibers; the majority was also positive for phospho-aSyn and for TH (Fig. 1B). The overall density of phospho-aSyn positive fibers in the striatum of aSyn-IF mice was about 50% lower than in aSyn-AL mice (Fig. 4E). The same difference was observed when we determined the ratio of phospho-aSyn and human aSyn positive fibers (Fig. 4F).

In order to validate this histological finding by a different approach, we measured the amounts of transduced human aSyn and endogenous rodent aSyn in protein lysates prepared from the SN. Triton X-100 fractionation allowed us to estimate the fraction of higher order aSyn species by calculating the ratio of signal intensity in the Triton X-100 insoluble fraction normalized by the Triton X-100 soluble fraction. Human aSyn was detected both in the Triton X-100 soluble fraction (Fig. 4G) and in the Triton X-100 insoluble fraction (Fig. 4H) of mice transduced with rAAV-aSyn, but not in lysates of animals transduced with rAAV-GFP. Strong accumulation of human aSyn in the Triton X-100 insoluble fraction was observed in aSyn-AL animals (Fig. 4H). This ratio was about 60% lower in aSyn-IF mice than in aSyn-AL mice (Fig. 4I), consistent with the reduced extent of aSyn pathology as reported by histology (Fig. 4D, F).

Rodent aSyn was also detected in the Triton X-100 insoluble fraction of mice transduced with rAAV-aSyn, but not in mice transduced with rAAV-GFP (Fig. 4H), suggesting that aSyn pathology involves not only the transduced human aSyn but also endogenous rodent aSyn. The fraction of Triton X-100 insoluble rodent aSyn was lower in lysates with IF diet than in mice with AL diet (Fig. 4H, J).

In summary, aSyn pathology in the SN was observed in mice transduced with rAAV-aSyn, and it was reduced by IF diet.

### Intermittent fasting shifts microglia phenotype

**Fig. 5:**
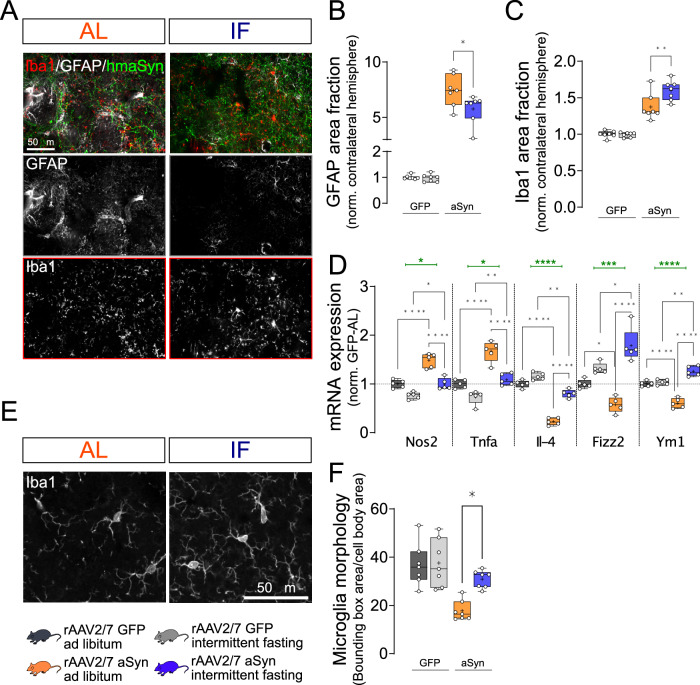
Intermittent fasting reduces aSyn-induced astroglia activation and shifts microglia polarization. **A** Representative images of ipsilateral striatal sections stained for GFAP (white in overlay images), Iba1 (red) and human aSyn (green). Scale bar: 50 μm. **B** Area fraction positive for GFAP signal, normalized to the (i) contralateral hemisphere of the same animal and to the (ii) rAAV-GFP–AL group. Box and whisker plots, middle line: median, +: mean, circles: individual animals; *n* = 7, two-way ANOVA, Tukey post-hoc test, *p* = 0.019. Area fractions from both hemispheres are on Supplementary Fig. 4A. **C** Area fraction positive for Iba1 signal, normalized to the (i) contralateral hemisphere of the same animal and to the (ii) rAAV-GFP–AL group. Box and whisker plots, middle line: median, +: mean, circles: individual animals; *n* = 7, two-way ANOVA, Tukey post-hoc test, *p* = 0.0075. Area fractions from both hemispheres are on Supplementary Fig. 4B. **D** Expression of *Nos2, Tnfa*, *Il-4, Fizz2, and Ym1* in the STR. Box and whisker plots, middle line: median, +: mean, circles: individual animals; *n* = 5, two-way ANOVA, Tukey post-hoc test (*Nos2*: ****: p < 0.0001; ++++: p < 0.0001; *: *p* = 0.0354; +: *p* = 0.0403) (*Tnfa*: ****: p < 0.0001; ++++: p < 0.0001; **: *p* = 0.00079; +: *p* = 0.0381) (*Il-4*: ****: p < 0.0001; ++++: p < 0.0001; **: *p* = 0.0044; ++++: p < 0.0001) (*Fizz2*: *: *p* = 0.012; ++++: p < 0.0001; +: *p* = 0.0232; ***: *p* = 0.00075) (*Ym1*: ****: p < 0.0001; ++++: p < 0.0001; **: *p* = 0.0085; ++++: p < 0.0001). Green line and * represent interaction between the treatments (vector and diet). **E** Representative images with larger magnification of microglia identified by staining for Iba1. Scale bar: 50 μm. Morphology of Iba1-positive cells was analyzed by comparing the ratio of the bounding box area to the area of the cell body (Supplementary Fig. 5C). **F** Quantification of “microglia morphology” (expressed as the ratio of the bounding box area and cell body area). Box and whisker plots, middle line: median, +: mean, circles: individual animals (minimum 50 cells per animal analyzed); *n* = 7, two-way ANOVA, Tukey post-hoc test. *p* = 0.0117. Box and whiskers plots: box: 25th to 75th percentiles, whiskers: from the smallest to the largest value. Source data are provided as a Source Data file.

Glial cells respond to toxic stimuli and neurodegeneration^45^, and may promote or inhibit pathological processes. We therefore quantified gliosis by staining for the astroglial marker GFAP and the microglia marker Iba1 (Fig. 5A). Striatal astroglia activation, measured as the area fraction positive for GFAP, was 7-fold higher in aSyn-AL mice compared to GFP-AL mice (Fig. 5B). In contrast, astroglia activation was less pronounced in aSyn-IF mice. IF did not affect astroglia in mice transduced with rAAV-GFP (Fig. 5B).

To assess microglia, we first quantified the Iba1-positive area fraction. It was 1.4-fold higher in aSyn-AL mice than in GFP-AL mice, and even higher in aSyn-IF mice (Fig. 5C). Activated microglia are characterized by a more compact and less branched morphology. To report microglia activation and process surveillance territory value, the ratio of the bounding box (connecting the distal-most edges of the microglial processes) area to the cell body size was calculated (Fig. 5E). Transduction of the SN with aSyn decreased the ratio of the bounding box area to the cell body area, suggesting microglia activation (Fig. 5F, representing cells with bounding boxes are on Supplementary Fig. 5C). This effect on microglia morphology was reverted by IF (Fig. 5F). Several damage-specific microglia subtypes have been described^46–48^. In aSyn-AL mice, expression of *Nos2* and *Tnfa*—as quantified by qPCR—was increased as compared to GFP-AL mice and the expression of *Il-4*, *Fizz2,* and *Ym1* was decreased (Fig. 5D). These changes are consistent with previous results by others^49,50^. All transcriptional changes were reduced by IF diet (Fig. 5D), consistent with the morphological findings (Fig. 5F). Indeed, microglia are regulated by metabolic state^51^, and the inflammatory response to LPS is blunted by IF^13^. Conversely, chronic activation of the innate immune system can be sufficient to trigger neurodegeneration^4^.

### Intermittent fasting reduces aSyn toxicity in aged mice

**Fig. 6:**
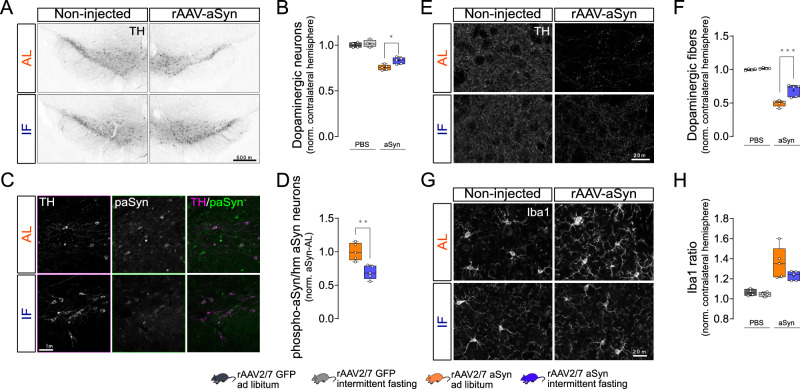
Intermittent fasting reduces aSyn-induced neurodegeneration in aged mice. **A** Representative images of midbrain sections from aged animals, stained for TH. Scale bar: 500 μm. **B** Normalized number of TH-positive neurons in the SN. Numbers were normalized to the (i) contralateral hemisphere and (ii) to the PBS–AL group. Box and whisker plots, middle line: median, +: mean, circles: individual animals; *n* = 4 for PBS and *n* = 5 for rAAV2/7-aSyn, two-way ANOVA, Tukey post-hoc test, *p* = 0.0134. **C** Representative images of the SN from aged animals unilaterally injected with rAAV2/7-aSyn vector, stained for TH (magenta pseudo color on overlay images) and phospho-aSyn (green). Scale bar: 50 µm. **D** Intensity of paSyn signal within human-aSyn-positive neurons, normalized to the rAAV-aSyn–AL group. Box and whisker plots, middle line: median, +: mean, circles: individual animals; *n* = 5, two-sided t-test, *p* = 0.003. **E** Representative images of striatal sections from aged animals, stained for TH. Scale bar: 20 μm. **F** Density of TH-positive fibers in the STR, normalized to the (i) contralateral hemisphere and (ii) to the PBS–AL group. Box and whisker plots, middle line: median, +: mean, circles: individual animals; *n* = 4 for PBS and *n* = 5 for rAAV2/7-aSyn, two-way ANOVA, Tukey post-hoc test, *p* = 0.0004). **G** Representative images showing Iba1 staining in the striatum of aged animals. Scale bar: 20 μm. **H** Area fraction positive for Iba1 signal, normalized to the (i) contralateral hemisphere of the same animal and to the (ii) PBS–AL group. Box and whisker plots, middle line: median, +: mean, circles: individual animals; *n* = 4 for PBS and *n* = 5 for rAAV2/7-aSyn, two-way ANOVA, Tukey post-hoc test. Data for GFAP is shown as Supplement to Fig. 6. Box and whiskers plots: box: 25th to 75th percentiles, whiskers: from the smallest to the largest value. Source data are provided as a Source Data file.

PD and synucleinopathies are age-dependent neurodegenerative diseases. We therefore wanted to confirm that IF is able to reduce aSyn-induced neurodegeneration in aged mice. 12–14 months old mice were unilaterally injected with rAAV-aSyn, and subjected to dietary intervention (Fig. 6). Eight weeks after surgery, rAAV-aSyn injection resulted in an ~25% reduction in the number of dopaminergic neurons in the SN in mice on AL diet (Fig. 6A, B), and ~50% reduction in the density of dopaminergic fibers in the striatum (Fig. 6E, F). Four weeks of IF reduced the loss of dopaminergic neurons to ~18%, and loss of striatal fibers to ~30%. In addition, IF reduced the staining intensity of phospho-aSyn (Fig. 6C, D), suggesting, that the accumulation of pathological aSyn species is reduced. Similar to young mice, IF did not affect microglia density in the STR (Fig. 6G, H).

In summary, IF was also able to reduce aSyn-induced neurodegeneration and aSyn pathology in aged mice—albeit to a lesser extent than in young animals.

### Intermittent fasting alters expression of genes related to aSyn-induced neuroinflammation in the ventral midbrain

**Fig. 7:**
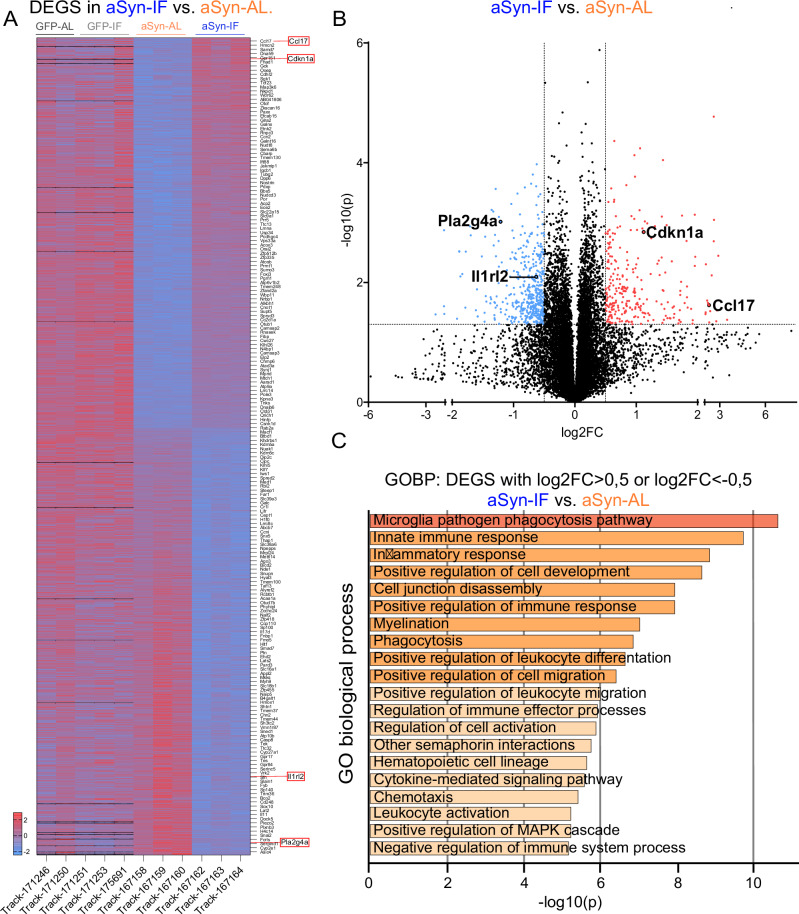
Intermittent fasting induces profound transcriptome changes in the ventral midbrain of mice injected with rAAV-aSyn. Bulk RNA sequencing was performed from the ventral midbrain of mice transduced with rAAV-GFP or rAAV-aSyn, and subjected to diet control. **A** Heatmap shows differentially expressed genes between aSyn-AL and aSyn-IF groups. Z-scores of genes of individual mice are used to generate the heatmap, note: only every 15th gene is labeled with names. **B** Volcano plot shows genes detected in aSyn-AL and aSyn-IF. Blue: down-regulated DEGs, red: up-regulated DEGs. For the graph, log2 fold change (log2FC) and log10 *p*-values were used. Genes are considered as DEG, when the log2FC > 0.5 or log2FC < −0.5, and log10(p) > 1.3 (p < 0.05). Genes selected for further validation are highlighted in (**A**, **B**). two-sided t-test, p < 0.05. **C** Gene Ontology (GO) analysis of DEGs. Genes, selected as in (**B**), were significantly regulated both in aSyn-AL vs. GFP-AL and aSyn-AL vs. aSyn-IF. Fisher’s exact test was used to compute the *p*-values and enrichment factors for each ontology category. Source data are provided as a Source Data file.

In order to obtain an unbiased picture about the effects of IF in the brain, we conducted a transcriptomic analysis of ventral midbrain samples obtained from mice transduced with rAAV-GFP or rAAV-aSyn and subjected to dietary intervention. In this analysis, we were mainly interested in genes expressed differentially (DEGs) between mice with aSyn transduction and IF vs. AL diet (Fig. 7A). To narrow down potential mechanisms altered by IF, we selected genes that were significantly regulated between (1) GFP-AL and aSyn-AL, and further significantly regulated between (2) aSyn-AL and aSyn-IF. Furthermore, the (3) log fold change (logFC) in the expression reached our criteria (logFC < −0.5 or logFC > 0.5) between aSyn-AL and aSyn-IF (Fig. 7B). Surprisingly, the most significantly regulated gene ontology (GO) biological processes were related to microglial activation, innate immune response and inflammatory response (Fig. 7C). This finding is in line with the modulation of microglia by metabolic state^51^, a blunted inflammatory response to LPS administration in the hippocampus after IF^13^ and neurodegeneration observed in models with chronic activation of the innate immune system^4^.

### Repeated nutrient deprivation reduces inflammatory signaling in primary neuronal cultures

In order to dissect further potential mediators of IF on aSyn-related neurodegeneration, we selected four targets from our transcriptomic screen. For this validation step, we chose molecules that can be manipulated by small molecules or replaced in cell culture. Two targets were upregulated in aSyn-IF compared to aSyn-AL (Fig. 6B), the cytokine C-C motif chemokine ligand 17 (*Ccl17*) and cyclin dependent kinase inhibitor 1A (*Cdkn1a);* two targets were downregulated, interleukin-1 receptor-like 2 (*l1rl2*) and the phospholipase A2 isoform group IVA (*Pla2g4a*). CCL17 can induce “M2”-like polarization on microglia^52–54^, IL1RL2 is the Toll-like receptor family member and IL-36 receptor that activates NF-κB^55^. PLA2G4A generates arachidonic acid and downstream lipid-based inflammatory mediators^56^. Cdkn1a is a cyclin-dependent kinase 2 inhibitor, also known as p21Cip1, a mediator between DNA damage response and neuronal senescence^57^.

**Fig. 8:**
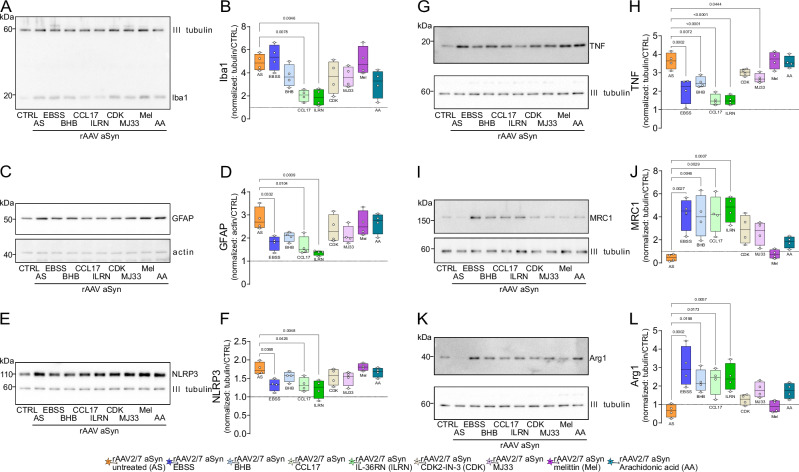
CCL17 and IL-36 inhibition reduces neuroinflammation in vitro. **A** Representative immunoblot showing βIII tubulin and Iba1 signal. **B** Ratio of the Iba1 and βIII tubulin signals, normalized to rAAV-GFP (CTRL). Box and whisker plots, middle line: median, +: mean, circles: individual preparations; *n* = 4, one-way ANOVA, Tukey post-hoc test. *p*-values are shown on the graph. **C** Representative immunoblots showing GFAP (upper panel) and βactin (lower panel) signals in primary cultures. **D** Ratio of the GFAP and βactin signals. Numbers were normalized to rAAV-GFP (CTRL). Box and whisker plots, middle line: median, +: mean, circles: individual preparations; *n* = 4, one-way ANOVA, Tukey post-hoc test. *p*-values are shown on the graph. **E** Representative immunoblot showing NLRP3 and βIII tubulin signals. **F** Ratio of the NLRP3 and βIII tubulin signals, normalized to rAAV-GFP (CTRL). Box and whisker plots, middle line: median, +: mean, circles: individual preparations; *n* = 4, one-way ANOVA, Tukey post-hoc test. *p*-values are shown on the graph. **G** Representative immunoblots showing TNFα (upper panel) and βIII tubulin (lower panel) signals. **H** Ratio of the TNFα and βIII tubulin signals, normalized to rAAV-GFP (CTRL). Box and whisker plots, middle line: median, +: mean, circles: individual preparations; *n* = 4, one-way ANOVA, Tukey post-hoc test. *p*-values are shown on the graph. **I** Representative immunoblots showing MRC1 (upper panel) and βIII tubulin (lower panel) signals. **J** Ratio of the MRC1 and βIII tubulin signals, normalized to rAAV-GFP (CTRL). Box and whisker plots, middle line: median, +: mean, circles: individual preparations; *n* = 4, one-way ANOVA, Tukey post-hoc test. *p*-values are shown on the graph. **K** Representative immunoblots showing Arg1 (upper panel) and βIII tubulin (lower panel) signals. **L** Ratio of the Arg1 and βIII tubulin signals, normalized to rAAV-GFP (CTRL). Box and whisker plots, middle line: median, +: mean, circles: individual preparations; *n* = 4, one-way ANOVA, Tukey post-hoc test. *p*-values are shown on the graph. Whole membranes are shown on Figure supplement for Fig. 7. Box and whiskers plots: box: 25th to 75th percentiles, whiskers: from the smallest to the largest value. Source data are provided as a Source Data file.

To validate the relevance of these regulations - increased *Ccl17* and *Cdkn1a* and decreased *Ilrl1* and *Pla2g4a* - we used mouse primary cultures containing neuronal and glial cells. rAAV-aSyn transduction induced glial responses and inflammatory signaling, as evidenced by increased levels of IBA1, GFAP, NLRP3, TNF, and decreased expression of MRC1 and ARG1 (Fig. 8A–L). The differential regulation of NLRP3 and TNF as opposed to MRC1 and ARG1 by rAAV-aSyn transduction is consistent with the differential regulation of microglia subtypes in mouse striatum observed by qPCR (Fig. 5D).

In order to simulate IF diet in vitro, primary cultures were kept in EBSS medium for 4 h in every 24 h, for 3 consecutive days. In addition, parallel cultures were exposed to BHB, which was increased in mouse blood during IF diet (Fig. 1B). Both treatments counteracted the aSyn-induced changes (Fig. 8A–L)—except that EBSS did not reduce the aSyn-induced increase in IBA1 expression (Fig. 8B). These findings are consistent with the reduction of aSyn-induced changes by IF diet in mice (Figs. 1–7) and with BHB mediating these effects. The effect of BHB but not EBSS on IBA1 expression is consistent with the modulation of microglia polarization by BHB^58–60^.

Treatment of rAAV-aSyn transduced cultures with either CCL17 or the interleukin receptor antagonist IL-36RN mitigated the aSyn-induced changes in neuroinflammation (Fig. 8A–L). The magnitude of changes was comparable to the effects of EBSS and BHB. In contrast to direct modulation of inflammatory processes, the small molecule inhibitor of CDK2, CDK2-IN-3, had only a moderate effect on aSyn-induced neuroinflammation (Fig. 8A–L).

Finally, we thought to validate the potential role of the PLA2 isoform *Pla2g4a* (Fig. 7B) by using the small molecule PLA2 inhibitor MJ33 and the PLA2 activator melittin. In addition, separate cultures were exposed to the PLA2 product arachidonic acid. Interestingly, PLA2 modulation had no strong effect on neuroinflammation in primary culture, with the exception of the reduction of TNF by MJ33 (Fig. 8H).

### Autophagy induction and reduced pro-inflammatory signaling are responsible for reduced aSyn pathology in primary cultures

**Fig. 9:**
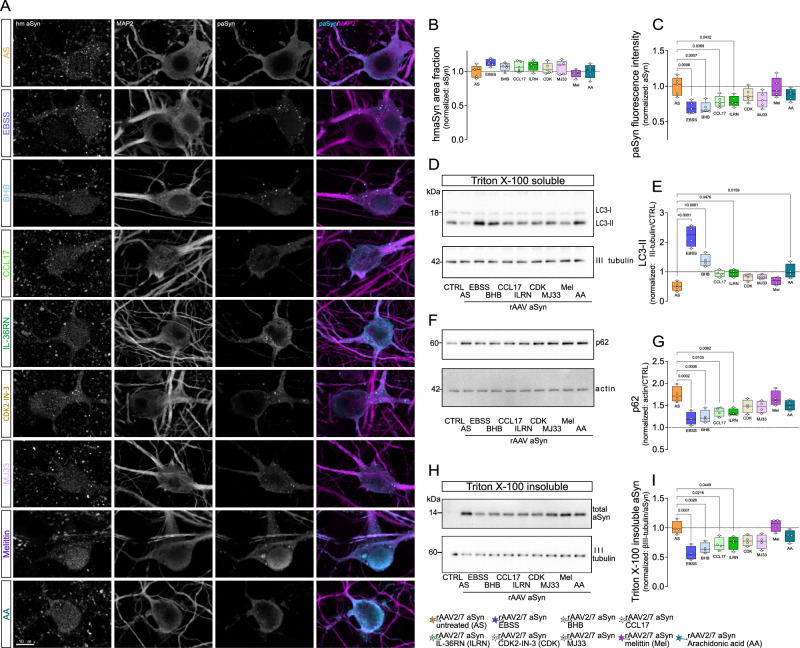
Reduced aSyn pathology in cultures with reduced inflammation. **A** Representative, high magnification images of primary neuronal cultures transduced with rAAV-aSyn and treated as indicated on the left side. Cultures were stained for human aSyn, MAP2 (magenta in overlay images), and phospho-aSyn (cyan in overlay images). Scale bar: 10 μm. **B** Area fraction of hmaSyn signal within MAP2-positive neurons, normalized to rAAV-aSyn control cultures. Box and whisker plots, middle line: median, +: mean, circles: individual preparations; *n* = 5, one-way ANOVA, Tukey post-hoc test. **C** Mean fluorescence intensity of paSyn signal within MAP2-positive neurons, normalized to rAAV-aSyn control cultures. Box and whisker plots, middle line: median, +: mean, circles: individual preparations; *n* = 5, one-way ANOVA, Tukey post-hoc test. *p*-values are shown on the graph. **D** Representative immunoblots showing LC3-I and LC3-II (upper panel) and βactin (lower panel) signals. **E** Ratio of the LC3-II (lipidated LC3, lower band) and βactin signals, normalized to rAAV-GFP (CTRL). Box and whisker plots, middle line: median, +: mean, circles: individual preparations; *n* = 4, one-way ANOVA, Tukey post-hoc test. *p*-values are shown on the graph. **F** Representative immunoblots showing SQSTM1/p62 (upper panel), βactin (upper band, lower panel) and aSyn (lower band, lower panel) signals. **G** Ratio of p62 and βactin signals, normalized to rAAV-GFP (CTRL). Box and whisker plots, middle line: median, +: mean, circles: individual preparations; *n* = 4, one-way ANOVA, Tukey post-hoc test. *p*-values are shown on the graph. **H** Representative immunoblots showing “total” aSyn (both human and rodent asyn) upper panel) and βIII tubulin (lower panel) signals in the Triton X-100 insoluble fraction. **I** Ratio of aSyn and βIII tubulin signals, normalized to the rAAV-aSyn untreated (rAAV aSyn CTRL) cultures. Box and whisker plots, middle line: median, circles: independent experiments/preparations; *n* = 4, one-way ANOVA followed by Tukey post-hoc test. *p*-values are indicated on the graph. Whole membranes are shown on Figure supplement 8. Box and whiskers plots: box: 25th to 75th percentiles, whiskers: from the smallest to the largest value. Source data are provided as a Source Data file.

In order to investigate the effects of the same manipulations on aSyn pathology in primary cultures, we stained for phospho-aSyn and analyzed levels of detergent insoluble aSyn by western blot. In addition, we monitored autophagy by analyzing LC3 and p62 levels. rAAV-aSyn transduction reduced the abundance of lipidated LC3 (LC3II, Fig. 9C), and increased the abundance of p62 (Fig. 9E), both suggesting decreased autophagy, consistent with previous findings^43^. In the rAAV-aSyn transduced cultures, levels of detergent-soluble aSyn were about threefold higher than in control cells and it was unaffected by treatments (Supplement for Fig. 9). EBSS increased LC3 lipidation (Fig. 9D, E) and reduced p62 accumulation (Fig. 9F, G), consistent with starvation-induced autophagy. BHB also increased LC3 lipidation and reduced p62 levels, consistent with previous findings^61,62^. The fact that both EBSS and BHB, a metabolic product accumulating during fasting periods, reduced the amount of detergent-insoluble aSyn (Fig. 9H) suggests, that our in vitro model recapitulates in vivo IF at least in part.

Interestingly, reducing pro-inflammatory signaling by treatment of cultures with either CCL17 or IL36RN, normalized the aSyn-induced reduction of LC3 lipidation (Fig. 9C), and reduced the accumulation of p62 and detergent-insoluble aSyn species (Fig. 9F, H). Arachidonic acid also normalized the aSyn-induced reduction of LC3II (Fig. 8C), but not p62 or detergent-insoluble aSyn levels. CDK2-IN-3, MJ33 and melittin did not induce significant effects.

### Inflammatory signaling reduces aSyn-induced toxicity in primary cultures

**Fig. 10:**
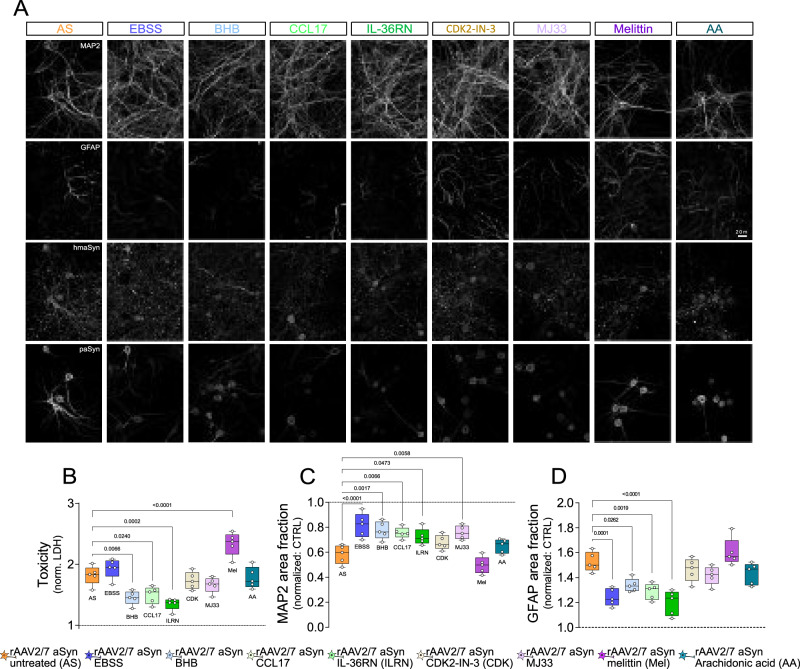
Anti-inflammatory treatment ameliorates aSyn toxicity in vitro. **A** Representative images of primary neuronal cultures transduced with rAAV-aSyn and treated as indicated on the top. Cultures were stained against MAP2, GFAP, human aSyn and p129S-aSyn. Scale bar: 20 µm. **B** LDH release in cultures treated as indicated and normalized to the positive (cultures treated with 1% Triton X-100) and negative (empty medium) controls, and for rAAV-GFP. Box and whisker plots, middle line: median, circles: independent experiments/preparations; *n* = 5, one-way ANOVA followed by Tukey post-hoc test. *p*-values are indicated on the graph. **C** Quantification of the area fraction positive for the MAP2-staining in rAAV-aSyn-transduced primary cultures, normalized to rAAV-GFP. Box and whisker plots, middle line: median, circles: independent experiments/preparations; *n* = 5, one-way ANOVA followed by Tukey post-hoc test. *p*-values are indicated on the graph. **D** Quantification of the area fraction positive GFAP, normalized for rAAV-GFP. Box and whisker plots, middle line: median, circles: independent experiments/preparations; *n* = 5, one-way ANOVA followed by Tukey post-hoc test. *p*-values are indicated on the graph. Data including the rAAV-GFP treated cultures is included as Supplement to Fig. 10. Box and whiskers plots: box: 25th to 75th percentiles, whiskers: from the smallest to the largest value. Source data are provided as a Source Data file.

Finally, we analyzed the effects of the described treatments on cellular integrity, by quantifying in primary mouse cultures the area positive for the neuronal marker MAP2 and the release of cytosolic LDH. aSyn transduction decreased MAP2 area (Fig. 10A, C) and increased LDH release (Fig. 10B). The decrease in MAP2 area was reversed by EBSS, BHB, CCL17, IL36RN and MJ33 (Fig. 10C). The aSyn-induced increase in LDH release was reversed by BHB, CCL17, IL36RN and melittin (Fig. 10C). Furthermore, we also found that reduction of pro-inflammatory signaling by IL36RN reduced cellular oxidative stress (Supplement for Fig. 10A), along with nitrite production (Supplement for Fig. 10B) and nuclear DNA damage (53BP1-positive foci, Supplement for Fig. 10C, D). The number of 53BP1-positive foci was also reduced in EBSS- and BHB-treated cultures.

## Discussion

In this study, we tested the potential of a dietary intervention to improve functional outcomes in an aSyn-based mouse model of Parkinson’s disease. Four weeks of intermittent fasting (IF) mitigated the aSyn-induced phenotype, which included degeneration of dopaminergic neurons, synuclein pathology and motor deficits.

In our model, rAAV2/7 vector-mediated overexpression of A53T aSyn in the SN induced an ~40% reduction in the number of dopaminergic neurons (Fig. 1E), 75% reduction in the striatal fiber density (Fig. 1F) and 60% reduction in striatal dopamine level (Fig. 2C), consistent with previous reports^20,63^.

Intermittent fasting (IF) was implemented as a dietary intervention. Specifically, animals were deprived of food for 24 h on non-consecutive days for 4 weeks. In order to mirror potential cases in patients, we first induced aSyn pathology and then, 4 weeks after rAAV injection, when aSyn pathology was already pronounced (Supplementary Fig. 1), IF regimen was initiated. Additionally, alternate day fasting is the most commonly used protocol of IF in mice^11,13,15,16^. IF reduced aSyn pathology (Fig. 4) and the motor phenotype (Fig. 2B, C). IF blunted the aSyn-induced degeneration of dopaminergic somata in the SN as well as the degeneration of dopaminergic fibers and synaptic puncta in the striatum (Figs. 1E, F, 2A, E, F). The effects of IF were confirmed in an independent set of experiments in aged mice (Fig. 6). The positive effects of IF are in accordance with the increasingly recognized role of metabolic factors in neurodegenerative diseases^64^. For instance, cognitive performance is worse and declines more rapidly in PD patients with diabetes mellitus^65,66^. In contrast, antidiabetic medication with the glucagon-like peptide-1 receptor agonist lixisenatide reduces motor progression over 12 months in patients with PD^67^.

One of the most profound effects of food deprivation is the induction of macroautophagy^9^. Indeed, IF regulated the autophago-lysosomal system in mice with aSyn overexpression (Fig. 3 and Supplementary for Fig. 3). In fact, the higher increase in LC3-positive area fraction in phospho-aSyn-containing neurons (~2.5-fold) compared to all cells (~1.2-fold) and the higher fraction of phospho-aSyn detected in LC3-positive vesicles suggest that autophagy is specifically activated in neurons overloaded with misfolded proteins. Possibly, repeated cycles of autophagy induction with IF diet are able to counteract the impairment of the autophagic machinery observed with aSyn overload (Fig. 9E, G), and enable neurons to better cope with the high intracellular amounts of aSyn.

In addition to inducing autophagy, IF has been shown to reduce neuroinflammation. Modulation of glial cells and inflammatory pathways by IF was previously observed in the hippocampus for instance in response to LPS^13^, in AD models^68,69^ and in intracerebral hemorrhage^70^. IF was also shown to directly affect microglia polarization and to reduce the production of pro-inflammatory markers, such as TNF or IL-1β^70^. In our study, IF did not only reduce the expression of pro-inflammatory markers, but it also increased the expression of genes related to anti-inflammatory processes and neurotrophic factors (Fig. 5, supplement for Fig. 2). IF-induced reduction of inflammatory markers has been described in healthy animals^71^. Yet, we did not detect significant changes in any of the features we analyzed. These findings suggest that the altered inflammatory response we observed in aSyn-IF mice is neither the mere consequence of reduced aSyn toxicity resulting from increased autophagic clearance, nor a direct effect of IF. Rather, IF appears to specifically reduce aSyn-induced neuroinflammation.

As previously reported, the levels of the ketone body BHB in blood was increased in mice on IF diet (Fig. 1B)^29,30^. Furthermore, consistent with the hypothesis that peripherally synthetized BHB could mediate some effects of the IF diet in the brain, BHB treatment blunted all consequences of rAAV-aSyn transduction in primary neurons (Figs. 8–10). Measuring the effects of IF on aSyn pathology in mice deficient for the BHB receptor, GPR109A/HCA2^27,60^, or keeping animals on ketogenic diet might confirm this hypothesis in the future.

In order to better understand the pathways responsible for the protective effects of IF against aSyn-induced neurotoxicity within the brain, we performed a transcriptomic analysis on the ventral midbrain (Fig. 7). Interestingly, the strongest transcriptional responses to IF were related to (neuro)inflammation (Fig. 7D), suggesting, that altering neuroinflammation is a fundamental component of IF-mediated neuroprotection against aSyn toxicity. A recent study showing that microglia activation promotes the propagation of aSyn, and reducing neuroinflammation with aspirin blocks the spreading of aSyn pathology^72^ further supports our findings. Indeed, our validation experiments confirmed, that CCL17 (upregulated by IF) and IL1RL2 (downregulated by IF) are both able to modulate aSyn pathology. Consistent with our in vivo and in vitro results, the chemokine CCL17 was shown to promote the polarization of microglia towards an anti-inflammatory phenotype in an intracerebral hemorrhage model^73^, alleviating neuronal apoptosis and neurobehavioral deficits^53^. IL1RL2 is a member of the IL-1 receptor family genes and its binding to IL-36 results in NF-kB pathway activation. Thus, its inhibition reduced aSyn-induced activation of pro-inflammatory signaling, including NLRP3 inflammasome activation and TNF production (Fig. 8).

Another target that we sought to further investigate is PLA2G4a. It was significantly up-regulated by aSyn overexpression, and its expression was normalized by IF (Fig. 7). Phospholipase A2 cleaves phospholipids releasing arachidonic acid (AA), an omega−6 fatty acid. As a dietary supplement, it might reduce risk of heart disease^74^, and is considered as a beneficial fatty acid, mediating the anti-inflammatory effects of fasting in the periphery^75^. However, in primary neuronal cultures, activation of PLA2 by melittin further increased aSyn-induced toxicity (Fig. 10). Furthermore, neither PLA2 activation nor its inhibition by MJ33, or AA had substantial effects on any feature we analyzed. The lack of effects of PLA2-modulation might be explained by the reduced complexity of the primary culture compared to the brain, and clarifying its role in IF-induced neuroprotection against aSyn-toxicity may require in vivo studies.

Although both autophagy induction and altered neuroinflammation can independently mitigate aSyn-induced toxicity, both pathways likely interact. For instance, the extensive autophagy in neurons overloaded with phospho-aSyn might partially be responsible for reduced pro-inflammatory signaling. Facilitated removal of damaged mitochondria reduces oxidative stress, including DNA damage (Supplement for Fig. 10). That, in turn, reduces cytosolic double-strand DNA–induced activation of stimulator of interferon genes (STING) and NLRP3, and downstream activation of innate immune response^2,76^. Furthermore, activated STING is degraded by autophagy^77^.

Taken together, our findings confirm the potential of IF and downstream mediators, such as BHB, CCL17 or IL-36R antagonist, to reduce aSyn pathology in this mouse model of PD. Mechanistically, we found the regulation of autophagy in phospho-aSyn-containing neurons by IF diet. In addition, we observed a modulation of inflammatory signals by IF diet in mice and a reduction of aSyn pathology by CCL17 and IL-36R inhibition in primary cultures. The bidirectional regulation between autophagy and inflammation deserves further exploration in subsequent studies and could lead to novel and improved therapies for PD.

## Methods

All animal experiments were carried out in accordance with the guidelines of the Federation for European Laboratory Animal Science Associations (FELASA) and approved by the Bioethical Committee of Landesdirektion Sachsen, Germany, project 25-5131/496/39.

Source of chemicals, antibodies, composition of buffers, equipment, and software used in this study are listed in Supplementary Table S1. Supplementary Table S2 provides an overview of the number of animals used in each experiment. A schematic representation of the timeline of treatments and sample collection is depicted in Fig. 1A. All primary antibodies used in this study were validated through experiments in which primary or secondary antibodies were omitted during staining. Only antibodies that did not produce false-negative signals in these tests were included.

### Recombinant AAV production and purification

rAAV of serotype 2/7 (rAAV 2/7) were produced in the Leuven Viral Vector Core, KU Leuven, Leuven, Belgium, as previously described^20,78^. rAAV encodes either the human A53T mutant of αSyn or enhanced green fluorescent protein (GFP) under the control of the CMVie enhanced synapsin1 promoter. Real-time PCR analysis was used for genomic copy (GC) determination.

### Animals and surgery

Animals were housed under a 12-h light and/or dark cycle with free access to pelleted food and tap water, and all cages were equipped with nesting material. All surgical procedures were performed using aseptic techniques. For experiments 1, 2, 3, and 6, eight- to 12-week-old male C57BL/6J mice were obtained from Charles River Laboratories (Sulzfeld, Germany). For experiment 4, transgenic mice of mixed gender expressing the autophagosome marker microtubule-associated protein 1A/1B light chain 3A (LC3) tagged by two fluorescent proteins, RFP and EGFP (C57BL/6-Tg(CAG-RFP/EGFP/Map1lc3b)1Hill/J) were obtained from The Jackson Laboratory (Bar Harbor, ME USA). They are referred to as tfl-LC3 mice from here on and were used previously by our group^38^. For experiment 5, 16–18-month-old female mice from our B6.Cg-Tg(Drd1a-tdTomato)6Calak/J colony were used.

For stereotactic injection of rAAV, mice were anaesthetized with intraperitoneal injection of ketamine (100 mg/kg) and xylazine (10 mg/kg). 2 × 1 µl of vector (rAAV A53T αSyn or rAAV GFP; total genome copies 5E + 9) was injected in the substantia nigra (SN, AP: −3; L: −1,2; DV: −4,3/4,1 from dura, using Bregma as reference) with a Hamilton syringe (Hamilton, Bonaduz, GR, Switzerland). For histology, mice were injected unilaterally to allow comparison with the uninjected hemisphere. For qPCR and protein analysis, mice were injected bilaterally.

In experiments 1–4 and 6, mice were randomly allocated into ad libitum (AL) or intermittent fasting (IF) groups 4 weeks after surgery. In experiments 1–3, mice received either rAAV-GFP or rAAV-aSyn injection (rAAV GFP-AL, rAAV GFP-IF, rAAV aSyn-AL, rAAV aSyn-IF). In experiments 4 and 6, mice received PBS instead of rAAV-GFP (PBS—AL, PBS—IF, rAAV aSyn—AL, rAAV aSyn—IF). IF was initiated at CET 15:00 (2 h prior to lights off) for 24 h on Mondays, Wednesdays, and Fridays (alternate day fasting for 3/7 days per week). Food access was controlled by transferring mice daily between fresh cages with or without food. Mice in the AL groups were also transferred between cages at the same time to standardize handling. All mice had free access to water throughout the study. Body weight was monitored daily (CET 14:00–15:00), starting 2 days before IF was introduced and throughout the 4-week course of alternate day IF diet. Blood glucose (tail vein, AccuCheck Glucometer, Roche, Germany) was controlled two times per week: after 24 h fasting, and after 24 h feeding on the following day. In experiment 5, IF was started 2 weeks after surgery and carried out for 10 weeks (12 weeks total).

In experiments 1-4, forelimb use was quantified by the cylinder test before surgery and every 2 weeks after surgery as previously^79,31^. In brief, contacts made by each forepaw with the wall of an 11-cm-wide clear glass cylinder were scored on videotapes by an observer blinded to the animals’ treatment group. The first 25 contacts were counted for each animal, and left forelimb usage was expressed as a percentage of all forelimb contacts. Control animals without lesion score around 50 % in this test. In addition, the overall time to complete the first 25 contacts was recorded (around 200 s in control animals). The vertical rod test was used to quantify movement dysfunctions as previously^79^. It was applied in three trials at each time point before the surgery and every 2 weeks after the surgery. In this test, mice were placed on top of a vertical rod (50 cm from ground) with their head oriented upwards. For each trial, the time required to orient themselves in a downward direction and to climb to the base of the rod was recorded. The three trials of each time point were averaged.

In experiments 1–4, 6, mice were sacrificed 8 weeks after surgery, i.e., after for weeks of IF or AL diet (Fig. 1A). In experiment 5, mice were sacrificed 12 weeks after surgery, i.e., after 10 weeks of IF or AL diet. For histology, mice were euthanatized with an overdose of ketamine (200 mg/kg, i.p.) followed by transcardial perfusion with 4% paraformaldehyde in phosphate buffered saline (PBS). After post-fixation (4% paraformaldehyde, 2 h) and cryoprotection (30% sucrose in PBS), 30 µm coronal brain sections were cut with a cryostat (Leica, Nussloch, Germany). Free-floating sections were stored at −20 °C in a cryopreserving solution (30% sucrose, 1% polyvinyl-pyrrolidone, 30% glycol in Tris buffer (TBS, pH 7.4)) until use. For protein, mRNA and HPLC analyses, mice were euthanatized by cervical dislocation. Brains were rapidly removed, washed in ice cold TBS and the striatum and SN were dissected. Tissue pieces were snap frozen in liquid nitrogen and stored at −80 °C until use. For BHB measurement, blood was collected from the heart, centrifuged after 30 min clotting at room temperature (2500 × *g*, 15 min), and serum was stored at −80 °C until use.

### Primary neuronal culture

Primary cortical cultures were prepared from E17 embryos of mixed sex, from NMRI mice as previously described^80^. Briefly, after dissection and trypsinization, dissociated neurons were plated onto 96-well plates suitable for imaging (25,000 cells per well), or onto 12-well plates for biochemical assays (200,000 cells per well), and maintained in Neurobasal medium containing 2% B27, 0.5 mM glutamax and antibiotics. One third of the medium was changed on every third day, and from the second medium change on, no antibiotics were added. On DIV 14, neurons were transduced with rAAV GFP or rAAV aSyn vector (final concentration for all 1.0e + 8 GC/ml). From DIV19, cultures were treated with different compounds (72 h) or were subjected to starvation (complete medium change to Earle’s Balanced Salt Solution (**EBSS**), 4 h). The 4-h starvation cycle was repeated on three consecutive days. Control cultures (CTRL) were incubated in complete Neurobasal medium supplemented with 0.1 % BSA. The following compounds were used: (1) **BHB** (2 mM); (2) **CCL17** (40 nM, supplemented with 0.1% bovine serum albumin (BSA) to prevent CCL17 from sticking to the plate); (3) **IL-36RN** (100 ng/ml, supplemented with 0.1 % BSA); (4) **CDK2-IN-3** (100 nM); (5) **MJ33** (40 µM); (6) **Melittin** (2 µM); **Arachidonic acid** (20 µM). Cells were fixed (4% paraformaldehyde, 10 min, 20 °C) on DIV22 for imaging, or lysed for western blotting. To measure cytotoxicity, cell culture medium (three wells per condition, left for 24 h after the last medium change) was used.

### Immunofluorescence staining

Immunofluorescent staining was performed in brain sections containing the SN or the striatum. In brief, free-floating sections were permeabilized and blocked using 0.3% Triton X-100 and 1% normal donkey serum in TBS for 1 h. Sections were incubated with the primary antibodies overnight at 4 °C and with fluorescently labeled secondary antibodies for 120 min at 20 °C (sources and dilutions of antibodies in Supplementary Table 1). Sections were mounted with Fluoromount-G.

To quantify the integrity of dopaminergic neurons and aSyn pathology, every fifth section throughout the entire SN or the entire striatum was stained for tyrosine hydroxylase (TH), aSyn phosphorylated at Serine 129 (phospho-aSyn, 11A5) and for human aSyn (15G4). To determine neuroinflammation, every sixth striatal section was stained for the astroglia marker glial fibrillary acidic protein (GFAP, ab4674) and for the microglia marker ionized calcium-binding adapter molecule 1 (Iba1, 019-19741). Lysosomes were stained with an antibody specific for the lysosomal marker lysosomal-associated membrane protein 1 (LAMP1). Synaptic density was assessed by staining every sixth section of the striatum against the presynaptic marker synapsin and the postsynaptic marker postsynaptic density protein 95 (PSD95).

To address neuronal integrity, astrogliosis, and aSyn pathology, fixed primary neurons were stained against microtubule-associated protein 2 (MAP2, ab225316), GFAP (ab4674), phospho-aSyn (ab51253), and anti-human aSyn (ALX-804-258). In brief, cells were permeabilized using 0.2% Triton X-100 in TBS (10 min). Non-specific sites were blocked by 2% BSA in TBS (RT, 1 h). Primary antibodies were applied in blocking solution (4 °C, overnight). Secondary antibodies were: Alexa 405 conjugated anti-chicken, Alexa 555 conjugated anti-rabbit, Alexa 647 conjugated anti-rat. Coverslips were mounted with Fluoromount G.

To detect DNA damage, primary cultures were stained for 53BP1 (PA5-54565), human αSyn (ALX-804-258), and MAP2 (ab225316). After incubation with secondary antibodies (Alexa 647 conjugated anti-rat, Alexa 555 conjugated anti-rabbit), sections were mounted with Fluoromount-G.

### Analysis of dopaminergic neurons, aSyn pathology, gliosis, and autophagy

The number of dopaminergic neuron somata in the *SN* was determined by supervised manual counting by an investigator blinded to the experimental groups as follows. For each animal, every fifth section throughout the rostro-caudal extent of the SN (2.54 to −3.88 mm posterior to Bregma based on Paxinos and Franklin, 2001) was incorporated into the counting procedure. In each section, mosaic images were acquired as z stacks (step size: 2 µm, 5 slices in total) from both hemispheres with a 20× objective (N.A 0.8, Axio Imager 2, Zeiss). Maximal intensity projection of z stacks was used (ImageJ 1.47 v). After adjusting the threshold and carefully marking the borders of the SN, and TH-positive cell bodies with a visible nucleus in the blue channel were counted using the Cell Counter plugin of ImageJ. The total number of TH-positive neurons per SN hemisphere was estimated by multiplying the counted cell number by five since every fifth section was assessed.

Gliosis was assessed on every sixth striatal section stained for GFAP and Iba1. Five images, randomly distributed across the entire striatum, were acquired per slice as z stacks using a 20x objective (step size: 2 µm, 5 slices in total NA 0,8, Spinning Disk microscope, Zeiss). Maximal intensity projection of z stacks was used (ImageJ). After adjusting the threshold for each channel, background subtraction (rolling ball radius 50) and noise removal (pixel size: 2) (ImageJ), the area fraction was determined using ImageJ from ten regions of interest per image. Results were analyzed using a generalized linear mixed model (glm) in RStudio (v 2.8.0) with a hierarchically nested design (animal, section, and image were set as random factors) as previously^31^.

The density of the dopaminergic axon terminals (“fibers”) in the striatum, the density of human-aSyn-positive structures and the density of phospho-aSyn-positive structures were determined as described previously^31,81^. In brief, every sixth section per animal, five images per section and ten boxes per image were analyzed in a hierarchically nested design as above. z-stack images were acquired (five planes, 0.3 µm step size, 40x objective, N.A. 0.95; Spinning Disk microscope, Zeiss). Maximal intensity projection of z stacks was used (ImageJ). Area fraction expressed as percent area was measured after noise removal and adjusting the threshold.

In tfl-LC3 mice, the area fraction of tfl-LC3, LAMP1-positive and phospho-aSyn positive structures were determined in the SN of mice transduced with rAAV encoding human A53T-aSyn. Images were acquired with a 40x objective (five planes, 0.3 µm step size, N.A. 0.95, Spinning Disk microscope, Zeiss). Maximal intensity projection of z stacks was used (ImageJ), and in each image, a mask was created based on the human-aSyn staining to select only the transduced neurons. Next, the signal of each channel was adjusted as follows: Brightness of the image was adjusted automatically, background (rolling ball radius 50) and noise (pixel size 1) were removed. Area fraction of the binarized signal in the RFP and GFP channels (for tfl-LC3) or for LAMP1 was measured within the human-aSyn positive neurons^82^.

To determine the subcellular position of acidic or neutral LC3-positive vesicles or phospho-aSyn positive puncta, we used the “shrink analysis” described previously^38^, with some modifications from the same images as above. First, cell bodies of neurons were manually outlined in ImageJ and saved as regions of interest (ROI). Then, by using the macro described in ref. ^38^, five ROIs with progressive 20% erosions of the outline were created. For each ring, the number of puncta was determined.

To determine neuronal viability, area fraction covered by the MAP2 signal was measured using ImageJ^4^. In brief, first the background was subtracted (rolling ball radius: 50), noise was removed (pixel size 1), Gaussian blur filter (radius: 2) was applied and finally images were binarized and area fraction was measured. The same MAP2 mask (as above) was used as ROI to measure area fraction of phospho-aSyn signal (see above) as well.

To determine neuronal integrity, area fraction of MAP2 was measured^31,82^. Transduction efficiency and phospho-aSyn pathology was addressed by determining area fraction of human aSyn-positive and phospho-aSyn-positive neurons, and mean fluorescent intensity of phospho-aSyn signal within the MAP2 positive neurons, as previously^31^.

To determine DNA double strand breaks, the number of 53BP1-positive neuronal nuclear loci was counted within MAP2-positive neurons transduced with human αSyn, as previously described^31,83^.

### Triton X-100 solubility

For aSyn protein quantification and detection of Triton X-100 insoluble proteins, snap-frozen SN tissue (app. 5 mg) or primary neurons from 12-well plate were mechanically homogenized in ice-cold phosphate buffered saline solution (0,1 M, pH 7,6). Triton X-100 was added to a final concentration of 1%, vortexed and incubated for 30 min at 4 °C. After centrifugation (14,000 × *g*, 30 min, 4 °C), supernatant was used as Triton X-100 soluble fraction. The pellet was washed in ice-cold PBS, centrifuged again and re-dissolved with sonication (10 s) in 50 µl buffer containing 2% SDS, 75 mM Tris, 15% glycerin, 3.75 mM EDTA pH 7.4, and protease inhibitors. Solution was briefly centrifuged (5 min, 14,000 × *g*, RT), and it was used as Triton X-100 insoluble fraction. 10 µg of Triton X-100 soluble lysate or 10 µl Triton X-100 insoluble fraction were loaded onto a 4–20% Tris/glycine SDS gel for western blot analysis. After blocking, membranes were incubated first in the presence of antibodies against human αSyn (ALX-804-258-L001), alpha-tubulin (ab6046), β-tubulin (PRB-435P), LC3 (2775), p62 (ab109012), NLRP3 (AG-20B-0014), Arg1 (610708), MRC1 (PA5-114370), TNF (PA1-40281), Iba1 (019-19741), rodent aSyn (4179), then with horse radish peroxidase-conjugated secondary antibodies (donkey anti-rat, goat anti-mouse and donkey anti-rabbit). Signal was visualized with chemiluminescent substrate and detected with Luminescent Image Analyzer. ImageJ software was used to determine the optical density of protein bands (Gels plugin). Data was normalized (1) to the expression of the tubulin loading control within line, and then (2) to the values in the control, GFP + AL animals, or CTRL cultures.

### BHB measurement

BHB levels were measured in serum (prepared as described above) by using a BHB assay kit (Sigma) according to the manufacturer’s protocol. In brief, serum was deproteinized with a 10 kDa filter (Amicon), reaction mixture was added to 15 µl cleaned serum per well in a clear 96-well plate (three technical replicates per animal), and absorbance was measured after 30 min incubation at 450 nm. Concentrations were calculated by using a BHB standard curve (provided by the kit).

### Toxicity and oxidative stress

To determine the cytotoxicity in primary cultures, extracellular concentration of LDH was measured from the medium collected before fixing the cells using the Cytotoxicity Detection Kit (Roche) according to the manufacturer’s protocol. Briefly, for each independent preparation and condition, three technical replicates were used and averaged. Absorbance was measured at 492 nm; reference wavelength was 620 nm. Background (medium) absorbance was subtracted from all values, and values were normalized both to control and to maximal lysed (treated with 2% Triton X-100, according to the protocol) cultures.

Cytoplasmic ROS was measured using dichlorodihydrofluorescein diacetate (DCFH-DA; 10 mM, 37 C, 30 min), as described previously^31,82^. Fluorescence at 485/530 nm was measured with plate reader.

The production of NO by iNOS was measured indirectly by assaying nitrites in the lysate using the Griess reaction^4^. In brief, 10 µg of lysates were incubated with equal amounts of Griess reagent (1% sulfanilamide, 0.1% naphthylethylenediamine in 2% phosphoric acid solution) for 20 min at room temperature. Absorbance was read at 550 nm.

### Quantitative Real Time PCR

Total RNA was extracted from snap frozen dissected SN and striatum using the RNeasy Mini Kit (Qiagen, Hilden, Germany) according to the manufacturer’s instructions. cDNA was generated by QuantiTect Reverse Transcription kit (Qiagen). Quantitative Real Time PCR was carried out using a Stratagene MX3000Pro system (Qiagen) and GoTaq®aPCR Master Mix with SYBR green fluorescence (Promega, Mannheim, Germany). Primer sequences are listed in Supplementary Table S2. Fold change expressions were calculated using the 2^−ΔΔCT^ method, with β-actin as the reference gene.

### HPLC

High-performance liquid chromatography was used to detect tissue dopamine levels from striatal samples. Brain tissue was mechanically homogenized in 0.1 N perchloric acid for 3 × 10 s at maximum speed on ice. After protein quantification (Pierce™ 660 nm Protein Assay; Multiskan™ FC, ThermoFisher Scientific™, Germany), the homogenates were centrifuged for 15 min (13,000 × *g*, 4 °C) and the supernatant run on a 1260 Infinity II Agilent system, column (ProntoSIL 120-C18-SH, VDS Optilab, Germany) at a flow rate of 0.22 ml/min and a column temperature of 35 °C before electrochemical detection (Decade Elite SenCell 2 mm, Antec Leyden, Netherlands). Mobile phase (pH 3.48 of 80 mM NaH2PO4*H2O, 0.5 mM EDTA-Di-Na, 0.72 mM sodium 1-octanesulfonate, H3PO4, and 2-Propanol). Sample peak areas were measured via Agilent OpenLAB CDS (Agilent, US).

### Transcriptomic analyses

Total RNA from the substantia nigra was extracted using the RNeasy Lipid Tissue Mini Kit (QIAGEN GmbH, Hilden, Germany). Poly-A enrichment was performed before library preparation using NEBNext Ultra II Directional RNA Library Prep Kit (New England Biolabs, Ipswich, USA). The RNA integrity number (RIN) was assessed using the RNA Nano 6000 Assay Kit of Bioanalyzer 2100 system (Agilent Technologies, CA, USA). RNA samples had a mean RIN = 8.6, all samples had RIN > 8. Samples were stored at −80 °C until further processing. 500 ng of total RNA was used for downstream RNA-seq applications. Fifty to sixty million paired end reads were generated for each sample on an Illumina NovaSeq 6000 (Illumina, San Diego, USA) at TUD’s Deep Sequencing Facility. Reads were mapped to the mouse genome GRCm39 UCSC M29 using Salmon^84^. Mapping rates were between 76 and 82 %. Sequencing reports were summarized using MultiQC^85^. Raw count data was imported into R version 4.3.1 using tximport version 1.28.0^86^. Differential gene expression analysis was performed using DESeq2 version 1.40.2^87^. Data visualization was performed with Metascape^88^ and GraphPad Prism (version 10.1.2). The complete datasets are uploaded to the GEO repository (GSE252874).

### Statistical analyses

“n” was set to the number of animals in each group (*n* = 7 for histology of WT animals, *n* = 4–5 for histology of tfl-LC3 animals, *n* = 5 for protein and metabolite analysis and for qPCR) or to the number of independent preparations for cell culture experiments. Sample sizes were determined with adequate statistical power on the basis of the literature and on previous data collected by our group (G*Power). Analyses were done blinded to the experimental conditions. On box (25th to 75th percentiles) and whisker (from the smallest value to the largest value) diagrams, middle line represents median, cross represents mean, markers represent individual animals; on bar graphs, markers represent individual animals, lines represent mean and standard deviation (SD). Data normality was tested by the Shapiro-Wilk test and graphically by QQ plot (R). t-test, one-way ANOVA or two-way ANOVA were performed using GraphPad Prism 9.0.0 or 10.1.2. Linear regression was performed using R (v 2.8.0). *P* values are indicated in the graphs by symbols with * or # representing *p* < 0.05, ** or ## representing *p* < 0.01, *** or ### representing *p* < 0.001. Exact *p* values are given in the Figure legend.

### Reporting summary

Further information on research design is available in the Nature Portfolio Reporting Summary linked to this article.

## Supplementary information

Supplementary Information

Reporting Summary

Transparent Peer Review file

## Source data

Source Data

## Data Availability

The transcriptomic dataset is publicly available at the GEO repository (GSE252872 and GSE252873). All other data supporting the conclusions of this article are included in the article and its additional files. Source data are provided with this paper.

## Code availability

The code used for analysis of the transcriptomic dataset is available as supplemental material.
