# Supplementary material for: Intermittent fasting reduces alpha-synuclein pathology and functional decline in a mouse model of Parkinson’s disease
Source: Nat Commun. 2025 May 14;16:4470. doi: 10.1038/s41467-025-59249-5 (PMC12078643; doi:10.1038/s41467-025-59249-5)
Supplement: Supplementary file 1 — Supplementary Information [file 41467_2025_59249_MOESM1_ESM.pdf]

Supplemental Material to:

Intermittent fasting reduces alpha-synuclein pathology and functional decline in a mouse model of Parkinson's disease

Éva M. Szegő[1,2,3*], Lennart Höfs[1,3], Anna Antoniou[4,5], Elisabeth Dinter[1,2], Nadine Bernhardt[6], Anja Schneider[2,4], Donato A. Di Monte[2], Björn H. Falkenburger[1,3#]

[1] Department of Neurology, TU Dresden, Dresden, Germany

[2] German Center for Neurodegenerative Diseases (DZNE), Bonn, Germany

[3] German Center for Neurodegenerative Diseases (DZNE), Dresden, Germany

[4] Department of Old Age Psychiatry and Cognitive Disorders, University Hospital Bonn, University of Bonn, Bonn, Germany

[5] Department of Pharmaceutical Sciences, University of Vienna, Vienna, Austria

[6] Department of Psychiatry and Psychotherapy, TU Dresden, Dresden, Germany

Table of Contents:

1. List of abbreviations
2. Supplemental Tables
3. Supplemental Figures
4. Code used for analysis of Transcriptomic data

1. List of abbreviations

AL	ad libitum
ALP	autophago-lysosomal pathway
AMPK	5' AMP-activated protein kinase
aSyn	alpha synuclein
Bdnf	brain-derived neurotrophic factor
BHB	beta-hydroxybutyric acid
Fizz2	resistin-like molecule beta/found in inflammatory zone 2
Gba	Lysosomal Acid Glucosylceramidase
Gdnf	glial cell-derived neurotrophic factor
GFAP	glial fibrillary acidic protein
GFP	green fluorescent protein
hm aSyn	human alpha synuclein
Iba1	ionized calcium-binding adapter molecule 1
IF	intermittent fasting
Igf1	insulin like growth factor 1
Il-4	interleukin-4
Irs1	insulin receptor substrate 1
LAMP1	lysosomal-associated membrane protein 1
LB	Lewy body
LC3	microtubule-associated proteins 1A/1B light chain 3B
LN	Lewy neurite
Mcoln	mucolipin-1, transient receptor potential cation channel, mucolipin subfamily, member 1
mTOR	mammalian target of rapamycin
Nos2	nitric oxide synthase 2 (inducible)
PD	Parkinson's disease
PGC-1α	peroxisome proliferator-activated receptor γ coactivator 1α
phospho-aSyn	phosphorylated (S129) alpha synuclein
PSD95	postsynaptic density protein 95
rAAV	adeno-associated viral vector
SN	substantia nigra
STR	striatum
Tfeb	transcription factor EB
TH	tyrosine hydroxylase
Tnfa	tumor necrosis factor
ULK1	Unc-51 like autophagy activating kinase 1
Ym1	chitinase-3-like

2. Supplemental Tables

Supplemental Table S1.– List of materials and antibody dilutions

Antibody	Dilution	Source	ID
Anti-Iba1, rabbit	1:1000	Fujifilm Wako Chemicals	Cat# 019-19741
Anti-GFAP, chicken	1:2000	Abcam	Cat# ab4674
Anti-Tubulin βIII, rabbit	1:5000	Covance	Cat# PRB-435P
Anti-phospho-aSyn, rabbit	1:1000	Abcam	Cat# ab51253
Anti-aSyn, human specific, rat	1:800	Enzo	Cat# ALX-804-258
Ant-aSyn, rodent specific, rabbit	1:1000	Cell Signaling	Cat# 4179
Anti-TH, sheep	1:2000	Pel Freez	Cat# P40101
Anti-PSD95, rabbit	1.1000	Synaptic Systems	Cat# 160002
Anti-LC3, rabbit	1:1000	Cell Signaling	Cat# 2775
Anti LAMP1, rabbit	1:1000	Cell Signaling	Cat# 9091
Anti-MAP2, chicken	1:4000	Abcam	Cat# ab5392
Anti-Synapsin, chicken	1:1000	Synaptic System	Cat# 106006
Anti-MAP2, A488 conjugated, rabbit	1:500	Abcam	Cat#: ab225316
Anti-53BP1, rabbit	1:1000	Thermo Fisher	Cat#: PA5-54565
Anti-SQSTM1/p62, rabbit	1:10000	Abcam	Cat#: ab109012
Anti-NLRP3, mouse	1:1000	AdipoGen	Cat#: AG-20B-0014
Anti-Arginase-1, mouse	1:2000	BD Biosciences	Cat#: 610708
Anti-CD206/MRC1, rabbit	1:300	Thermo Fisher	Cat#: PA5-114370
Anti-TNF, rabbit	1:1000	Thermo Fisher	Cat#: PA1-40281
HRP conjugated donkey anti-mouse IgG	1:5000	Jackson ImmunoResearch	Cat# 715-035-150
HRP conjugated donkey anti-rabbit IgG	1:5000	Jackson ImmunoResearch	Cat# 711-035-152
HRP conjugated goat anti-mouse IgG	1:5000	Bio-Rad	Cat#: 1706516
Alexa 555 conjugated donkey anti-rabbit	1:2000	Invitrogen	Cat# A31572
Alexa 647 conjugated donkey anti-chicken	1:2000	Jackson ImmunoResearch	Cat# 703-605-155
Alexa 488 conjugated donkey anti-sheep	1:2000	Invitrogen	Cat# A11015
Alexa 488 conjugated donkey anti-rabbit	1:2000	Invitrogen	Cat# A21206

**Reagents and kits**			
PhosSTOP phosphatase inhibitor		Sigma-Aldrich	Cat# 4906845001
Bromphenol blue		Honeywell Fluka	Cat# 32712
Mini-PROTEAN TGX Precast Gels 4-20%		BioRad	Cat# 4561094
Precision Plus Protein WesternC Protein Standards		BioRad	Cat# 1610376
WesternBright Chemiluminescence Substrate Sirius		Biozym Scientific GmbH	Cat# 541019
Fluoromount-G		Southern Biotech	Cat# 0100-01
LDH Cytotoxicity Detection kit		Merck	Cat# 4744926001
BHB Assay kit		Sigma	Cat# MAK041
RNeasy Mini Kit		Qiagen	Cat# 74104
QuantiTect Reverse Transcription kit		Qiagen	Cat# 205313
GoTaq®aPCR Master Mix		Promega	Cat# A6102
RNeasy Lipid Tissue Mini Kit		Qiagen	Cat#: 74804
NEBNext Ultra II Directional RNA Library Prep Kit		New England Biolabs	Cat#: E7760
CCL17	40 nM	Medchem Express	Cat# HY-P71891A
IL-36RN	100 ng/ml	MedChem Express	Cat# HY-P77007
CDK2-IN-3	100 nM	MedChem Express	Cat# HY-112460
MJ33	40 µM	Santa Cruz	Cat# sc-221947
Melittin	2 µM	MedChem Express	Cat# HY-P0233

**Equipment**	
Infinite 200M Multimode-Plate-Reader	Tecan Group
CM3050S Kryostat	Leica Mikrosysteme Vertrieb GmbH
Zeiss Axio Observer Z1	Carl Zeiss
Zeiss Spinning Disc	Carl Zeiss
**Software**	
GraphPad Prism 5. 01 and 9.5.0	GraphPad Software
ImageJ Fiji, 1.47v	Wayne Rasband (NIH)

Supplemental Table S2.– List of animal experiments

Exp.	Genotype	Age	Injection	Number /group	Diet	Diet/post-OP survival	Analyses	Figures
1	WT	8-12 weeks	Unilateral, rAAV-GFP or rAAV-aSyn	n=4	only AL	4 weeks	Histology	Supl. Fig 1F-H
2	WT	8-12 weeks	Unilateral, rAAV-GFP or rAAV-aSyn	n=7	AL, IF 4 weeks	8 weeks	Behaviour[1], histology	Fig1, 2, 4 Supl. Fig1, 2, 3, 5
3	WT	8-12 weeks	Unilateral, rAAV-GFP or rAAV-aSyn	n=5	AL, IF 4 weeks	8 weeks	Behaviour[1], WB, qPCR, HPLC	
4	tfl-LC3	8-12 weeks	Unilateral, PBS or rAAV-aSyn	n=4-5	AL, IF 4 weeks	8 weeks	Behaviour[1], histology	Fig3, Supl. Fig3
5	WT	48-56 weeks	Unilateral, PBS or rAAV-aSyn	n=4-5	AL, IF 4 weeks	8 weeks	Behaviour[1], histology	Supl. Fig1-6
6	WT	8-12 weeks	Unilateral, rAAV-GFP or rAAV-aSyn	n=3	AL, IF 10 weeks	12 weeks	Behaviour[1], transcriptomics	Fig6

3. [1] Only the behaviour data from experiment 2 are shown in Figure 2.

Supplemental Table S3.– List of qPCR primers

Gene	Forward primer	Reverse primer
Ym1	CATTCAGTCAGTTATCAGATTCC	AGTGAGTAGCAGCCTTGG
Fizz2	TGGAGAATAAGGTCAAGGAAC	GTCAACGAGTAAGCACAGG
Il4	AGATGGATGTGCCAAACGTCCTCA	AATATGCGAAGCACCTTGGAAGCC
Tnfa	TTCCGAATTCACTGGAGCCTCGAA	TGCACCTCAGGGAAGAATCTGGAA
Nos2	CTGCTGGTGGTGACAAGCACATTT	ATGTCATGAGCAAAGGCGCAGAAC
Bdnf	GGCTGACACTTTTGAGCACGTC	CTCCAAAGGCACTTGACTGCTG
Gdnf	CCTTCGCGCTGACCAGTGACT	GCCGCTTGTTTATCTGGTGACC
Tfeb	CAGCAGGTGGTGAAGCAAGAGT	TCCAGGTGATGGAACGGAGACT
Lamp1	CCAGGCTTTCAAGGTGGACAGT	GGTAGGCAATGAGGACGATGAG
Mtor	AGAAGGGTCTCCAAGGACGACT	GCAGGACACAAAGGCAGCATTG
Igf1	GTGGATGCTCTTCAGTTCGTGTG	TCCAGTCTCCTCAGATCACAGC
Irs1	TGTCACCCAGTGGTAGTTGCTC	CTCTCAACAGGAGGTTTGGCATG
Mcolm1	GTCGGTGTCATTCGCTACCTGA	GAACGATCCAGCCACAGAAGCA
Actin	TGTGATGGTGGGAATGGGTCAGAA	TGTGGTGCCAGATCTTCTCCATGT
SNCA	ACCAAACAGGGTGTGGCAGAAG	CTTGCTCTTTGGTCTTCTCAGCC
GFP	AAGCAGAAGAACGGCATCAA	GGGGGTGTTCTGCTGGTAGT

3. Supplemental Figures

Supplement to Figure 1.

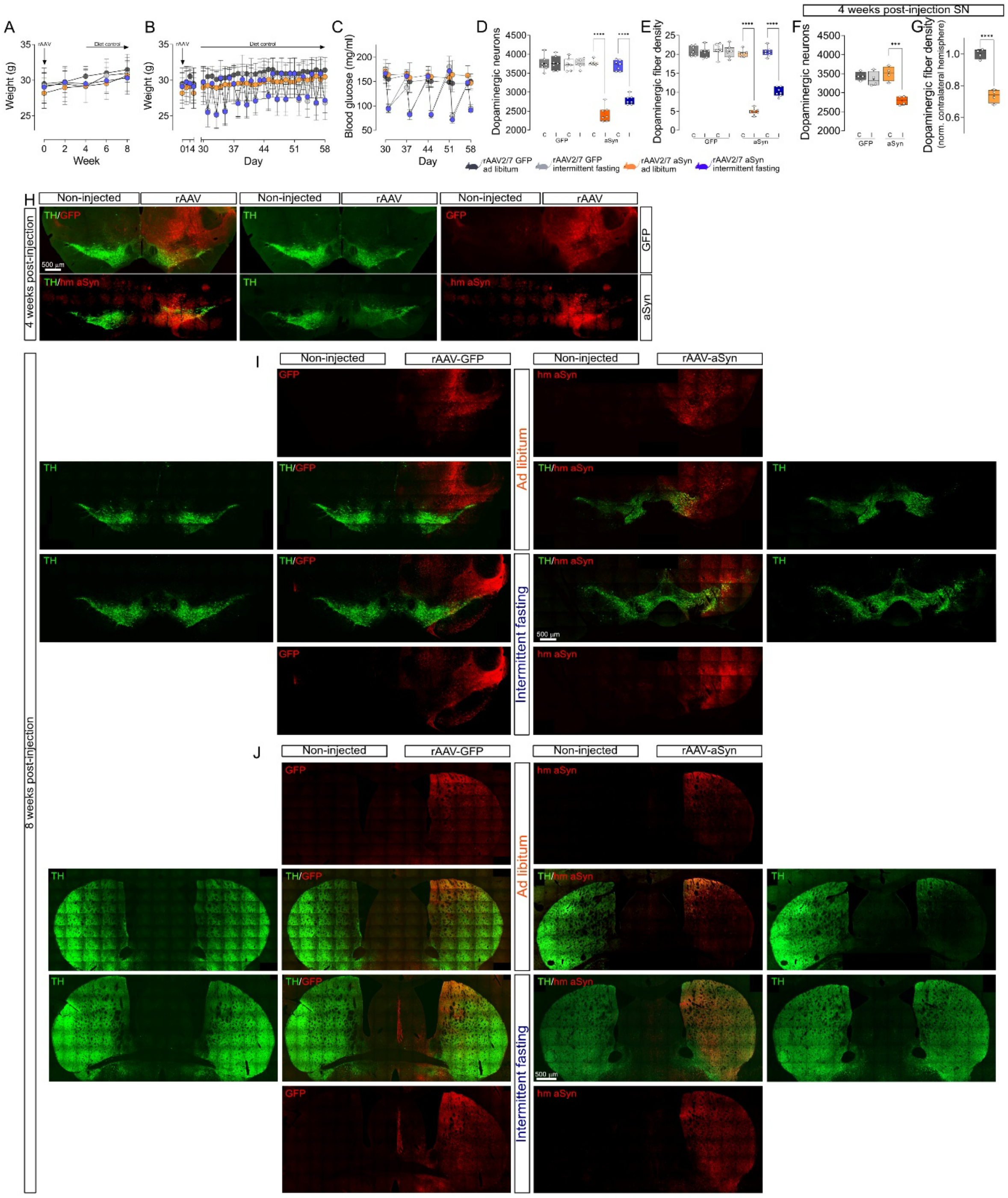

A) Biweekly measured body weight (g), measured after 24 h feeding. Mean ± SD, n=12, repeated measures ANOVA, Tukey post-hoc test. B) Daily change in the body weight (g) of mice during the diet control. Mean ± SD, n=12, repeated measures ANOVA, Tukey post-hoc test. C) Weekly measured blood glucose concentration (mg/ml) of mice during the diet control, measured after 24 h feeding and 24 h fasting. Mean ± SD, n=12, repeated measures ANOVA, Tukey post-hoc test. D) Number of dopaminergic neurons in the SN contralateral (C) and

ipsilateral (I) to injection. Box and whisker plots, middle line: median, +: mean, circles: individual animals; n=7, two-way ANOVA, Tukey post-hoc test, ****: p<0,0001. E) Density of dopaminergic fibers in the STR contralateral (C) and ipsilateral (I) to the injection. Box and whisker plots, middle line: median, +: mean, circles: individual animals; n=7, two-way ANOVA, Tukey post-hoc test, ****: p<0,0001. F) Number of dopaminergic neurons in the SN contralateral (C) and ipsilateral (I) to the vector injection, four weeks after surgery. Box and whisker plots, middle line: median, +: mean, circles: individual animals; n=4, two-way ANOVA, Tukey post-hoc test, p = 0.003. G) Density of dopaminergic fibers in the STR contralateral (C) and ipsilateral (I) to the injection, four weeks after surgery. Box and whisker plots, middle line: median, +: mean, circles: individual animals; n=4, two-way ANOVA, Tukey post-hoc test, ****: p < 0.0001. H) Representative images of midbrain sections, four weeks after the surgery. Sections were stained for TH (green) and for human aSyn or GFP (red). Scale bar: 500 µm. I) Representative images of midbrain sections, eight weeks after the. Sections were stained for TH (green) and for human aSyn or GFP (red). Scale bar: 500 µm. J) Representative images of striatal sections, eight weeks after the surgery. Sections were stained for TH (green) and for human aSyn or GFP (red). Scale bar: 500 µm. Box and whiskers plots: box: 25th to 75th percentiles, whiskers: from the smallest to the largest value. Source data are provided as a Source Data file.

Supplement to Figure 2.

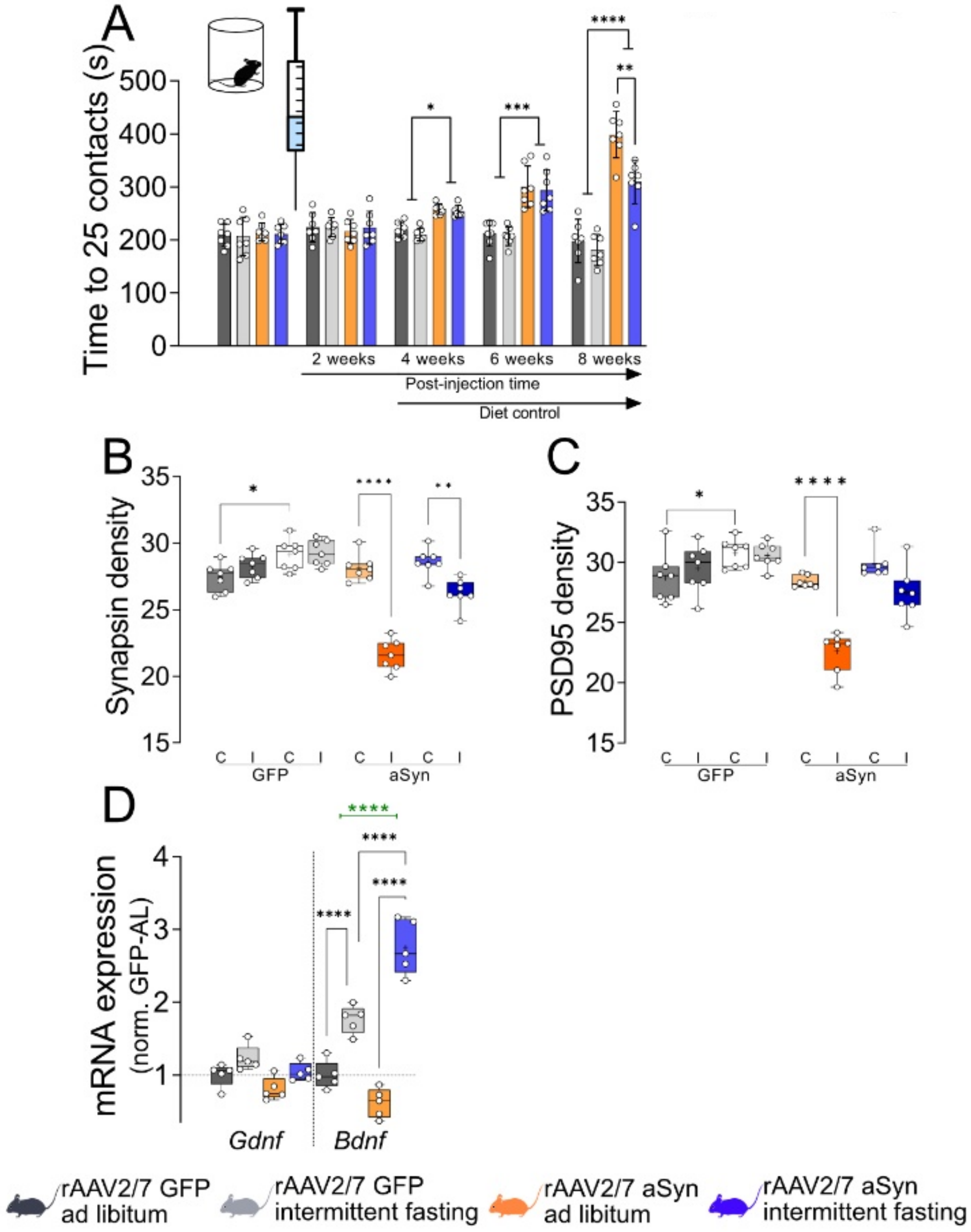

A) Quantification of time (s) until the first 25 paw contacts using the cylinder test. Mice were tested before and two, four, six and eight weeks after the vector injection. Mean ± SD, circles: individual animals; n=7, repeated measures ANOVA, Tukey post-hoc test; * p=0,0127; *** p=0,00083; ** p=0,0089; ****: p<0,0001. B) Density of synapsin-positive structures in the STR contralateral (C) and ipsilateral (I) to the vector injection (measured as area fraction). Box

and whisker plots, middle line: median, +: mean, circles: individual animals; n=7, two-way ANOVA, Tukey post-hoc test, ****: p<0,0001, **: p=0,0062; *: p = 0,0076. C) Density of PSD95-positive structures in the STR contralateral (C) and ipsilateral (I) to the vector injection (measured as area fraction). Box and whisker plots, middle line: median, +: mean, circles: individual animals; n=7, two-way ANOVA, Tukey post-hoc test, ****: p<0,0001; *: p = 0,0326. D) Expression of *Gdnf* and *Bdnf* in the striatum. Box and whisker plots, middle line: median, +: mean, circles: individual animals; n=7, two-way ANOVA, Tukey post-hoc test, ****: p<0,0001, green line indicates interactions (genotype and diet). Box and whiskers plots: box: 25th to 75th percentiles, whiskers: from the smallest to the largest value. Source data are provided as a Source Data file. Schemes were created by BioRender.

Supplement to Figure 3.

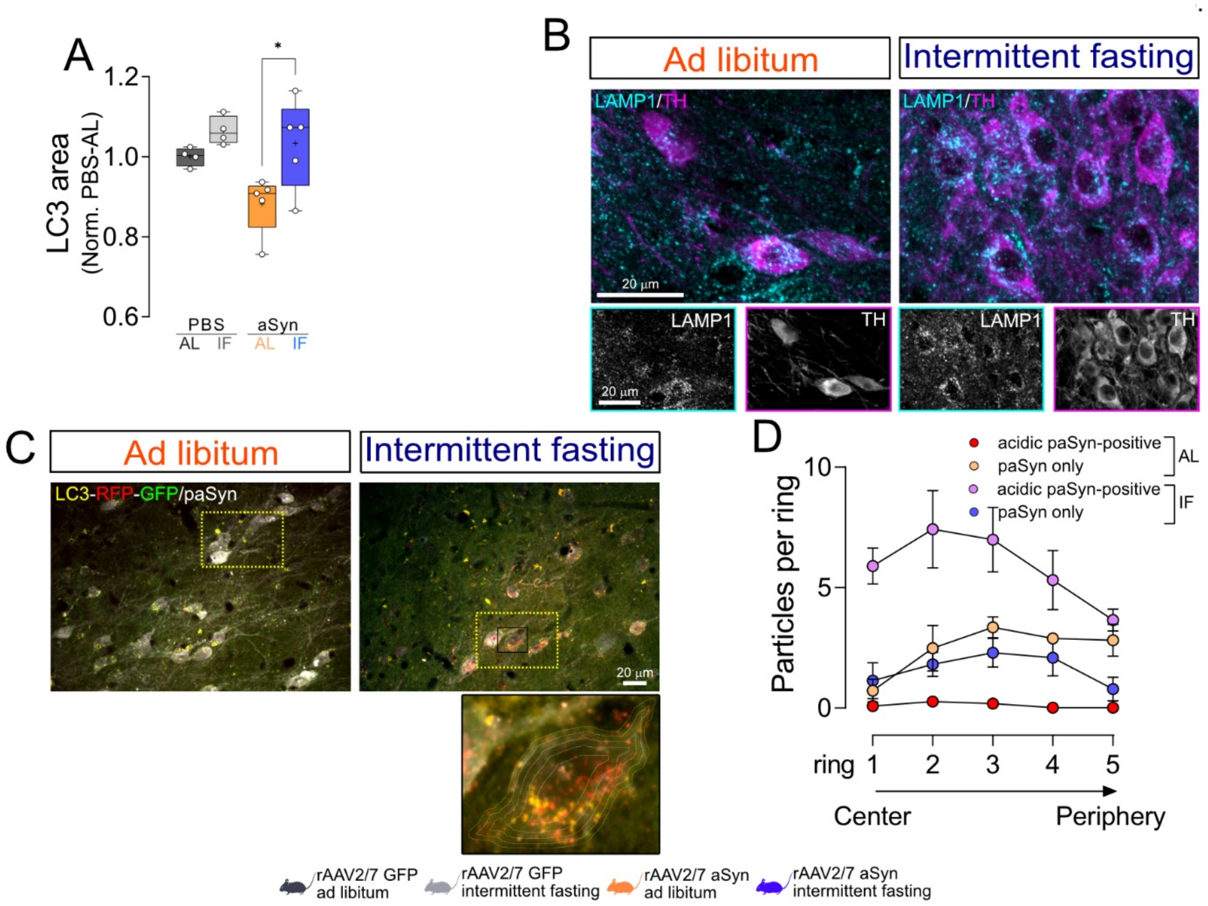

A) Area fraction covered by LC3 signal (RFP channel) in the SN (measured as area fraction). Box and whisker plots, middle line: median, +: mean, circles: individual animals; n=4 fr PBS and n=5 for rAAN-aSyn, two-way ANOVA, Tukey post-hoc test, *: p=0,028. B) Representative images of SN sections of rAAV-aSyn-injected tfl-LC3 animals stained for LAMP1 (cyan) and TH (magenta). Scale bar: 20µm. Lower images: individual channels (gray). C) Representative, low magnification images from the SN of LC3-tfl animals, showing LC3 (red and green) and phospho-aSyn (white) signal. Marked area is shown on Figure 5. D) Average number of acidic, phospho-aSyn-positive (red and purple) vesicles and structures positive only for phospho-aSyn (orange and blue) in each ring based on [23]. Mean ± SD, two-way ANOVA, Tukey post-hoc test. Box and whiskers plots: box: 25th to 75th percentiles, whiskers: from the smallest to the largest value. Source data are provided as a Source Data file. Schemes were created by BioRender.

Supplement to Figure 5.

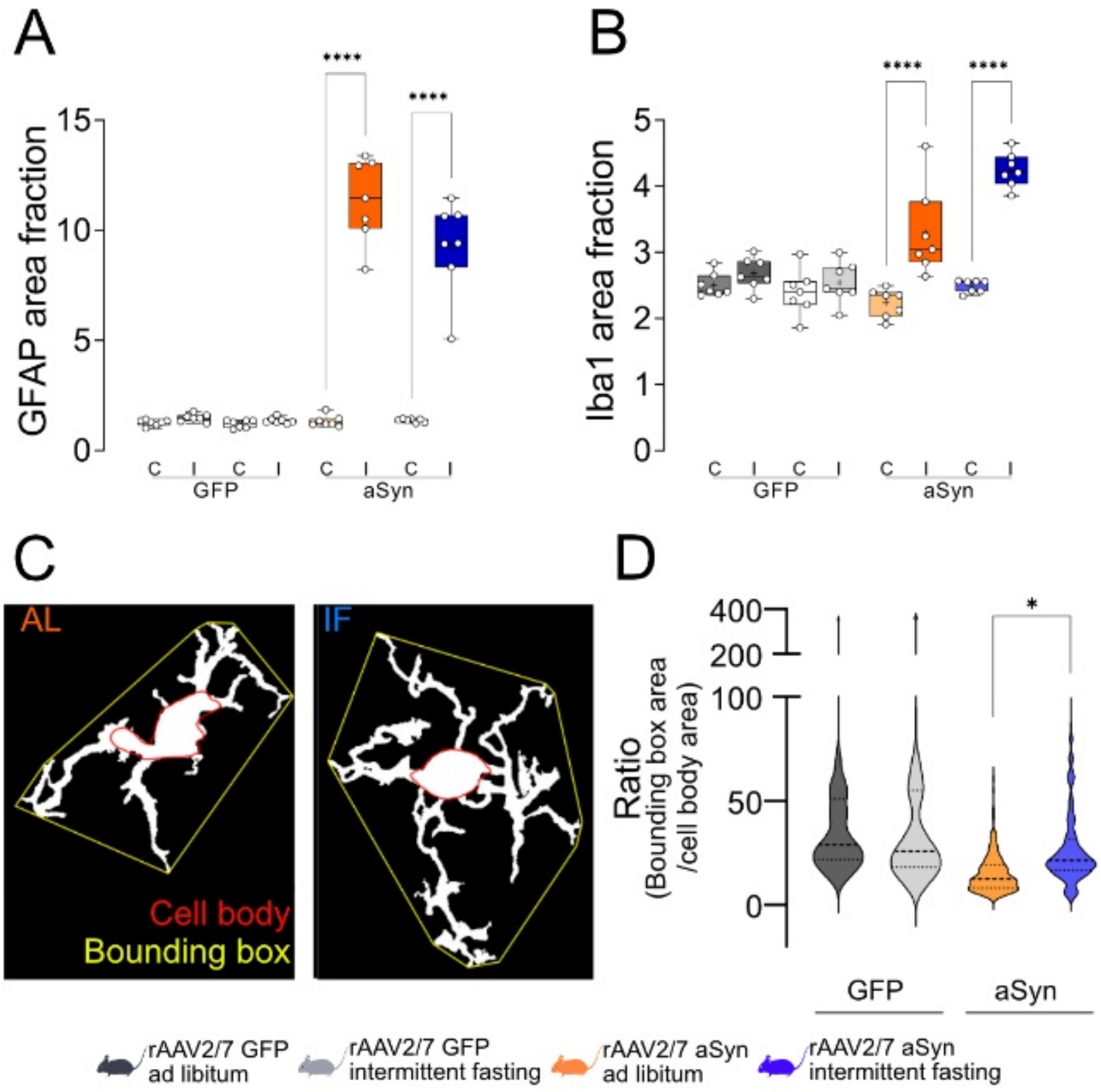

A) Area fraction covered by the GFAP signal in the STR contralateral (C) and ipsilateral (I) to the vector injection (measured as area fraction). Graph shows box and whisker plots, middle line: median, +: mean, circles: individual animals; n=7, two-way ANOVA, Tukey post-hoc test, ****: p<0,0001. B) Area fraction covered by the Iba1 signal in the STR contralateral (C) and ipsilateral (I) to the vector injection (measured as area fraction). Graph shows box and whisker plots, middle line: median, +: mean, circles: individual animals; n=7, two-way ANOVA, Tukey post-hoc test, ****: p<0,0001. C) Illustration how the bounding box and the cell area were determined for analysis of microglia cell morphology. D) Violin plot of the ratio for bounding box area / cell body area for all minimum 50 cells per animal (n=7 mice) are included in the analysis. Two-way ANOVA, Tukey post-hoc test, p=0,012, factors: rAAV, diet, animal. Source data are provided as a Source Data file. Schemes were created by BioRender.

Supplement to Figure 6.

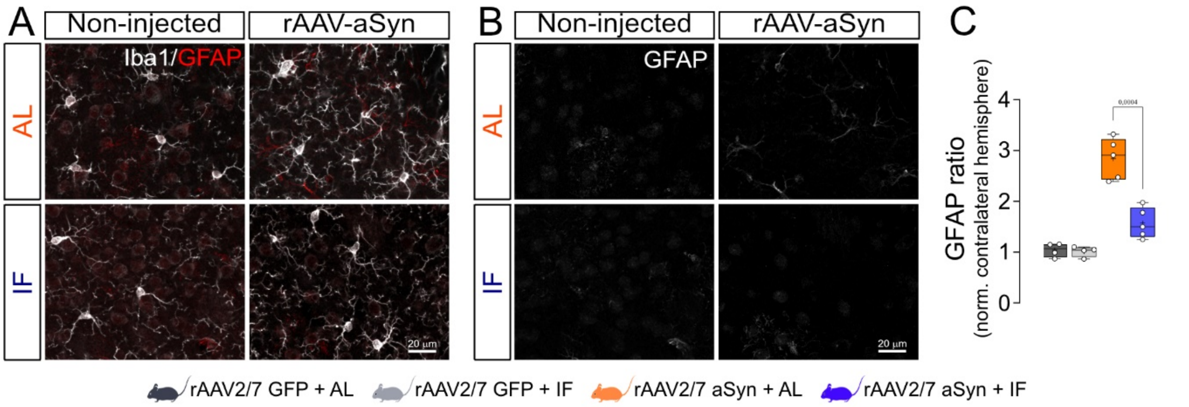

A) Representative images showing Iba1 (gray pseudocolor) and GFAP (red) staining in the striatum of aged animals. Scale bar: 20µm. B) Representative images showing only GFAP staining in the striatum of aged animals, as in panel (A). Scale bar: 20µm. CAarea fraction positive for GFAP signal, normalized to the i) contralateral hemisphere of the same animal and to the ii) PBS–AL group. Box and whisker plots, middle line: median, +: mean, circles: individual animals; n=4 (PBS) or n=5 (rAAVaSyn), two-way ANOVA, Tukey post-hoc test. Box and whiskers plots: box: 25th to 75th percentiles, whiskers: from the smallest to the largest value. Source data are provided as a Source Data file. Schemes were created by BioRender.

Supplement to Figure 8.

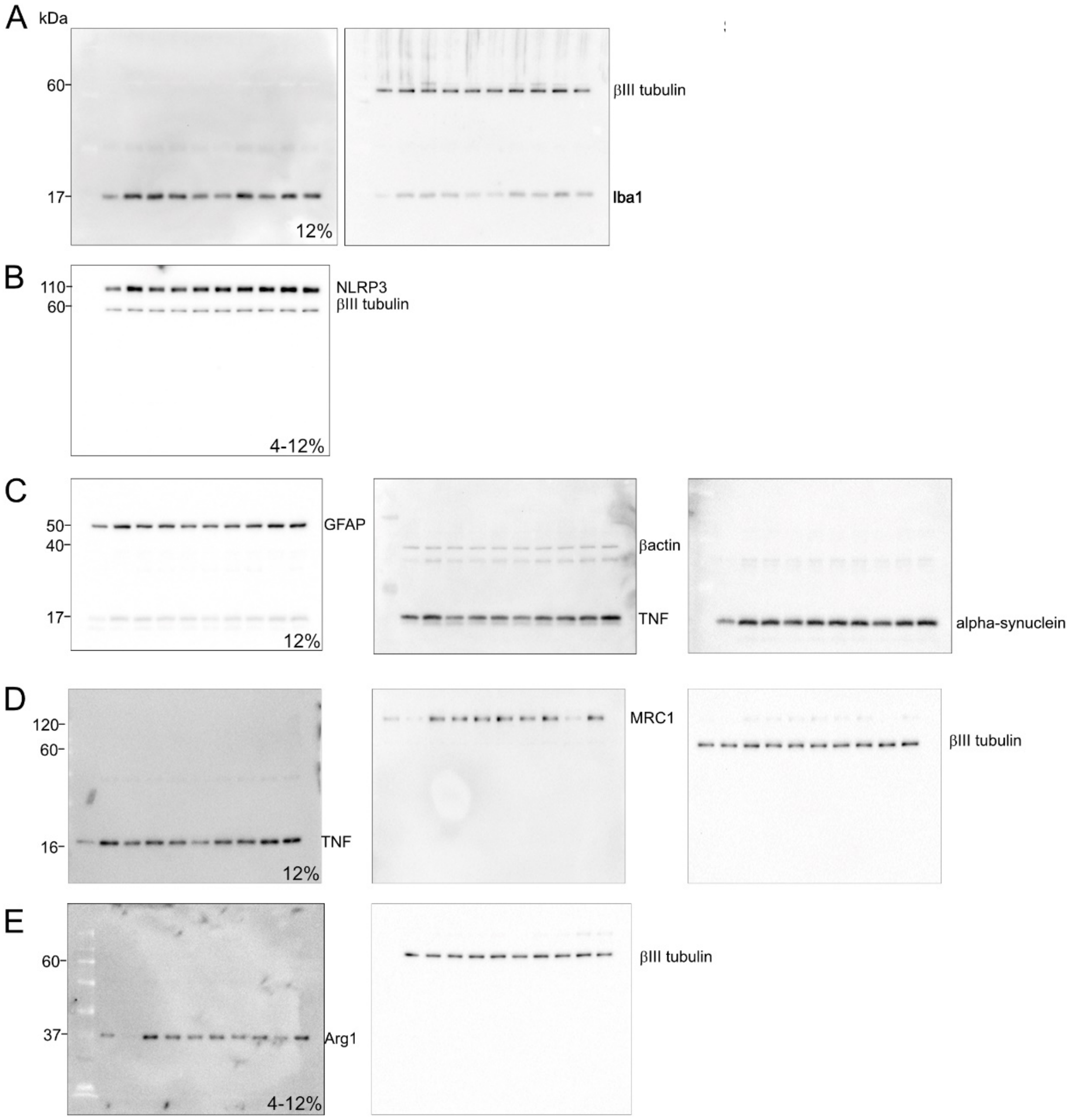

Uncropped membranes from Figure 8.

Supplement to Figure 9.

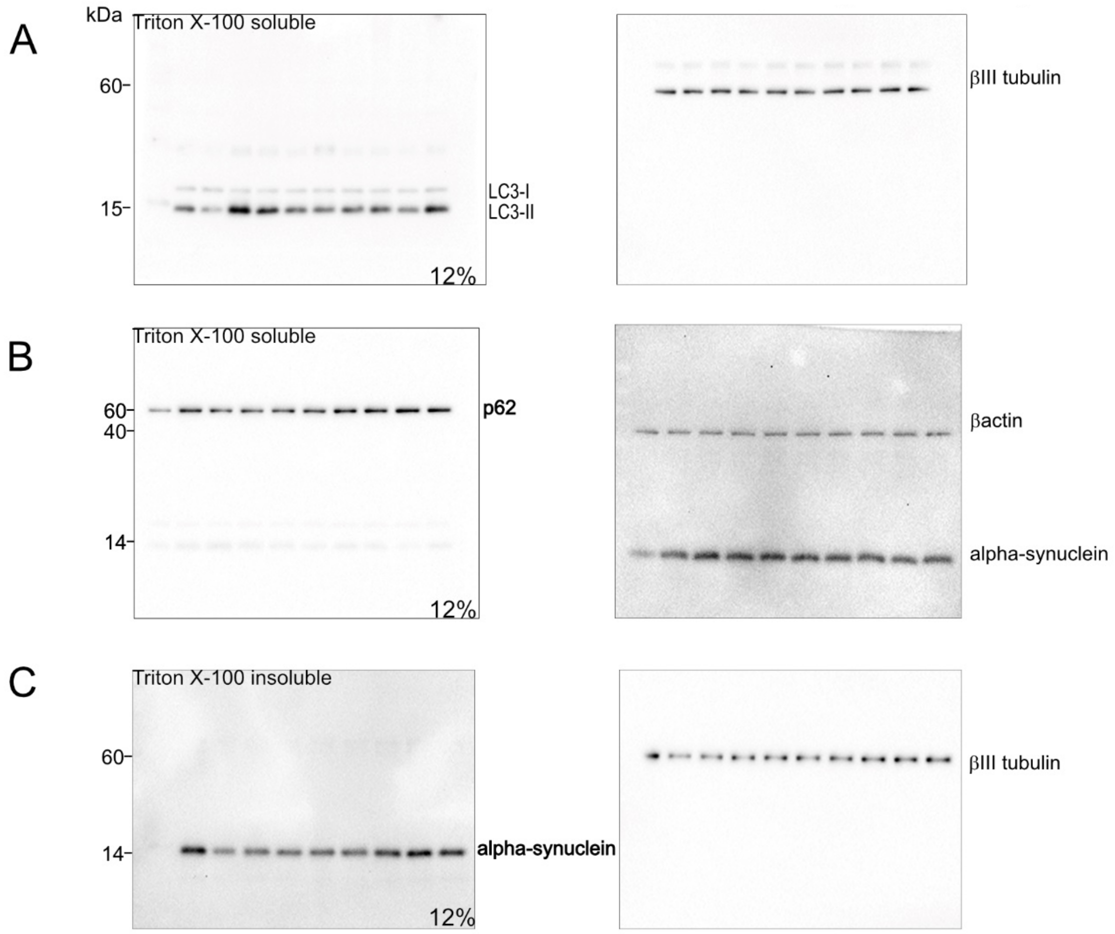

Uncropped membranes from Figure 9.

Supplement to Figure 10.

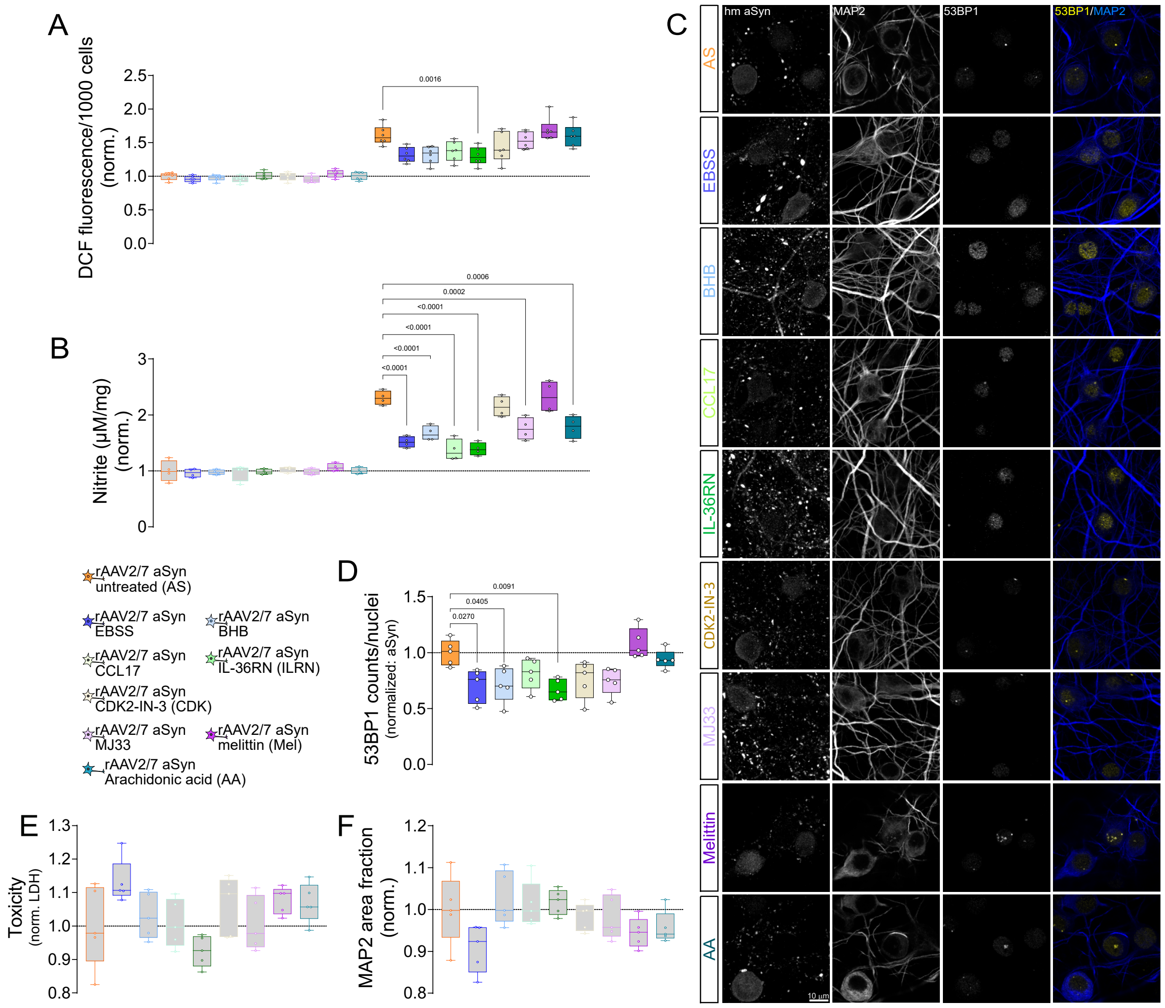

A) Quantification of DCFH-DA fluorescent signal expressed relative to the values measured from the cultures transduced with rAAV-GFP. B) Nitrite levels as markers of nitric oxide activity measured in lysates of primary cultures transduced with rAAV-aSyn. Values are normalized to the mean of rAAV-GFP transduced cultures. C) Representative images of primary neurons stained for the DNA damage response protein 53BP1 (yellow on the composite image), MAP2 (blue on the composite image) and human aSyn. Images show 53BP1-positive foci in the nuclei representing DNA damage sites. Scale bar: 10µm. D) Mean number of 53BP1-foci per neuronal nucleus (MAP2-positive cells), normalized to non-treated rAAV-aSyn transduced cultures. Graphs show box and whisker plots, middle line: median, circles: individual preparations; n=4-6, one-way ANOVA, Tukey’s post-hoc test. p-values are indicated above the connecting lines. E) Quantifications of toxicity (LDH release, normalized to the positive (cultures treated with 1% Triton X-100) and negative (empty medium) controls, and also for the rAAV-GFP control culture) in primary cultures transduced with rAAV-GFP. Data from cultures transduced with rAAV-aSyn are presented on Figure 10. Treatment with the

compounds was optimized to induce no significant cell death in control, rAAV-GFP cultures. Graphs show box and whisker plots, middle line: median, circles: individual preparations; n=5, two-way ANOVA, Tukey’s post-hoc test. F) Quantification of the area fraction positive for MAP2-staining in rAAV-GFP-transduced primary cultures. Numbers were normalized to the non-treated rAAV-GFP cultures. Data from cultures transduced with rAAV-aSyn are presented on Figure 10. Graphs show box and whisker plots, middle line: median, circles: individual preparations; n=5, two-way ANOVA, Tukey’s post-hoc test. Box and whiskers plots: box: 25th to 75th percentiles, whiskers: from the smallest to the largest value. Source data are provided as a Source Data file.A) Representative images of primary neuronal cultures transduced with rAAV-aSyn and treated as indicated on the left. Cultures were stained against GFAP, MAP2, p129S-aSyn and human aSyn. Scale bar: 20 µm. B) Quantification of the area fraction positive for the MAP2-staining in rAAV-aSyn-transduced primary cultures. Numbers were normalized to the control rAAV-GFP group. C) LDH release was measured in cultures treated as indicated and normalized to the positive (cultures treated with 1% Triton X-100) and negative (empty medium) controls, and also for the rAAV-GFP control culture. D) Quantification of the area fraction positive for the human aSyn-staining in rAAV-aSyn-transduced primary cultures. Numbers were normalized to the non-treated rAAV-aSyn cultures. E) Quantification of the fluorescence signal intensity of p129S-aSyn-staining within MAP2-positive neurons in rAAV-aSyn-transduced primary cultures. Numbers were normalized to the non-treated rAAV-aSyn cultures. Graphs show box and whisker plots, middle line: median, box: 25th and 75th percentiles, whiskers: range, circles: individual preparations; n=5, one-way ANOVA, Tukey post-hoc test. p-values are indicated above the connecting lines. Data including the rAAV-GFP treated cultures is included as Supplement to Figure 10.

4. Code for transcriptomic analysis

```
# plotting RNAseq data from Lennart of the fasting project

library(tidyr)
library(dplyr)
library(ggplot2)
library(hablar)
library(stringr)

# load data
setwd("~/Downloads/")
asyn <- read.table(file = "RNAseq_fasting_asyn_norm.csv", header = TRUE, na.strings = "",
sep = ",", dec = ".")
wt <- read.table(file = "RNAseq_fasting_wt_norm.csv", header = TRUE, na.strings = "", sep =
",", dec = ".")
metadata <- read.table(file = "RNAseq_fasting_metadata.csv", header = TRUE, na.strings =
"", sep = ",", dec = ".")
head(metadata)

# pivot and merge with metadata
head(asyn)
asyn <- subset(asyn, select=-X)
asyn <- subset(asyn, select=-gene_id)
asyn_long <- pivot_longer(asyn, cols=!external_gene_name, names_to = "track_ID",
names_prefix = "Track.", values_to = "reads", values_drop_na = FALSE)
head(asyn_long)

wt <- subset(wt, select=-X)
wt <- subset(wt, select=-gene_id)
wt_long <- pivot_longer(wt, cols=!external_gene_name, names_to = "track_ID",
names_prefix = "Track.", values_to = "reads", values_drop_na = FALSE)
head(wt_long)

metadata <- subset(metadata, select=-X)
metadata2 <- separate(metadata, col=File, into=c(NA, "track_ID"), sep="-")
head(metadata2)

wt_long2 <- merge(wt_long, metadata2)
head(wt_long2)

asyn_long2 <- merge(asyn_long, metadata2)
head(asyn_long2)

##### definitions
# canonical
canonical = c("Aif1","P2ry12","Tmem119","Cx3cr1","Cxcr4","Cd9","Lpl","Trem2")
asyn_long2$canonical <- asyn_long2$external_gene_name %in% canonical
wt_long2$canonical <- wt_long2$external_gene_name %in% canonical
```

```
m1 = c("Cd86","Tnf","H2-Ab1","Cd68","Ifng","Nos2","Nos1","Isg15","Chil3","Il1b","Fcgr3",
    "Cxcl10","Il6","Irf1","Stat1")
asyn_long2$m1 <- asyn_long2$external_gene_name %in% m1
wt_long2$m1 <- wt_long2$external_gene_name %in% m1

m2 = c("Arg1","Il10","Il13","Bdnf","Gdnf","Cd163","Cd206","Mrc1","Tgfb1","Chil3","Retnla",
    "Ngf")
asyn_long2$m2 <- asyn_long2$external_gene_name %in% m2
wt_long2$m2 <- wt_long2$external_gene_name %in% m2

bdnf =
c("Ntrk2","Ntrk2a","Ntrk2b","Bdnf","Plg","Plat","Mecp2","Syt4","Slc2a4","Slc9a6","Rapgef2"
,"Tiam1","Fstl4","Fam72a")
asyn_long2$bdnf <- asyn_long2$external_gene_name %in% bdnf
wt_long2$bdnf <- wt_long2$external_gene_name %in% bdnf

bdnf2 = c("Ntrk2","Bdnf","Plat","Mecp2","Syt4","Slc2a4","Slc9a6","Tiam1","Fstl4")
asyn_long2$bdnf <- asyn_long2$external_gene_name %in% bdnf2
wt_long2$bdnf <- wt_long2$external_gene_name %in% bdnf2

microglia_activation=c("Ager","Aif1","App","Atm","C1qa","C5ar1","Casp1","Clu","Csf1r","Cst
7","Ctsc",
              "Cxc3cl1","Cx3cr1","Grn","Hspa4","Ifng","Ifngr1","Il4","Il13","Il33","Itgam",

"Jak2","Jun","Kcnj8","Ldlr","Lrrk2","Mir7116","Mmp8","Naglu","Nampt","Nr1d1",
              "Pparg","Snca","Sphk1","Stap1","Syt11","Tafa3","Tlr1","Tlr2","Tlr3","Tlr4",
              "Tlr6","Tlr9","Tnf","Trem2","Trpv1","Ttbk1","Tyrobp")
asyn_long2$microglia_activation <- asyn_long2$external_gene_name %in%
microglia_activation
wt_long2$microglia_activation <- wt_long2$external_gene_name %in% microglia_activation

astroglia_activation =c("Adora2a","Ager","Agt","App","C1qa","C5ar1","Cntf","Csf1r","Egfr",
              "Grn","Ifng","Ifngr1","Il1b","Ldlr","Lrp1","Naglu","Nr1d1","Psen","Smo",
              "Trem2","Zeb2")
asyn_long2$astroglia_activation <- asyn_long2$external_gene_name %in%
astroglia_activation
wt_long2$astroglia_activation <- wt_long2$external_gene_name %in% astroglia_activation

autophagy =
stringr::str_to_title(tolower(c("ULK1","ULK2","BECN1","ATG5","ATG7","ATG12","ATG16L1","
SQSTM1","LC3","WIPI1","MTOR","ULK3","TFEB","ATG4B")))
asyn_long2$autophagy <- asyn_long2$external_gene_name %in% autophagy
wt_long2$autophagy <- wt_long2$external_gene_name %in% autophagy

autophagy2 = stringr::str_to_title(tolower(c("ATG5","ATG7","WIPI1","TFEB","ATG4B")))
wt_long2$autophagy <- wt_long2$external_gene_name %in% autophagy2
```

```
head(asyn_long2)
head(wt_long2)

# make plots for asyn
asyn_plot <- asyn_long2[which(asyn_long2$canonical==TRUE), ]
asyn_plot <- asyn_long2[which(asyn_long2$m1==TRUE), ]
asyn_plot <- asyn_long2[which(asyn_long2$m2==TRUE), ]
asyn_plot <- asyn_long2[which(asyn_long2$bdnf==TRUE), ]
p <- ggplot(asyn_plot, aes(x=group, y=reads, color=group)) +
  geom_dotplot(binaxis="y", stackdir="center") +
  facet_grid(cols=vars(external_gene_name), scales="free", space='free') +
  theme_classic() +
  theme(strip.text.x = element_text(angle = 90))
p


#### normalize to median of al"
head(asyn_long2)
df_grouped <- group_by(asyn_long2, external_gene_name)
amount_class <- summarise(df_grouped, median_gene = median(reads),  )
head(amount_class)
asyn_long2 <- merge(asyn_long2, amount_class)
asyn_long2$norm_reads <- asyn_long2$reads / asyn_long2$median_gene

head(wt_long2)
df_grouped <- group_by(wt_long2, external_gene_name)
amount_class <- summarise(df_grouped, median_gene = median(reads),  )
head(amount_class)
wt_long2 <- merge(wt_long2, amount_class)
wt_long2$norm_reads <- wt_long2$reads / wt_long2$median_gene
head(wt_long2)


# make plots for asyn
asyn_plot <- asyn_long2[which(asyn_long2$canonical==TRUE), ]
asyn_plot <- asyn_long2[which(asyn_long2$m1==TRUE), ]
asyn_plot <- asyn_long2[which(asyn_long2$m2==TRUE), ]
asyn_plot <- asyn_long2[which(asyn_long2$bdnf==TRUE), ]

p <- ggplot(asyn_plot, aes(x=group, y=norm_reads, color=group)) +
  geom_violin() +
  geom_dotplot(binaxis="y", stackdir="center") +
  facet_grid(cols=vars(external_gene_name), scales="free", space='free') +
  theme_classic() +
  theme(axis.text.x = element_text(angle = 90))
p
```

```
asyn_plot <- asyn_long2[which(asyn_long2$microglia_activation==TRUE), ]
asyn_plot <- asyn_long2[which(asyn_long2$astroglia_activation==TRUE), ]
asyn_plot <- asyn_plot[which(asyn_plot$external_gene_name != "Adora2a"), ]
asyn_plot <- asyn_long2[which(asyn_long2$autophagy==TRUE), ]

p <- ggplot(asyn_plot, aes(x=external_gene_name, y=norm_reads, color=group)) +
  geom_violin() +
  geom_dotplot(binaxis="y", stackdir="center") +
  facet_grid(cols=vars(group), scales="free", space='free') +
  theme_classic() +
  theme(axis.text.x = element_text(angle = 90))
p


# make plots for wt
wt_plot <- wt_long2[which(wt_long2$canonical==TRUE), ]
wt_plot <- wt_long2[which(wt_long2$m1==TRUE), ]
wt_plot <- wt_long2[which(wt_long2$m2==TRUE), ]
wt_plot <- wt_long2[which(wt_long2$bdnf==TRUE), ]

p <- ggplot(wt_plot, aes(x=group, y=norm_reads, color=group)) +
  geom_violin() +
  geom_dotplot(binaxis="y", stackdir="center") +
  facet_grid(cols=vars(external_gene_name), scales="free", space='free') +
  theme_classic() +
  theme(axis.text.x = element_text(angle = 90))
p

wt_plot <- wt_long2[which(wt_long2$microglia_activation==TRUE), ]
wt_plot <- wt_long2[which(wt_long2$astroglia_activation==TRUE), ]
wt_plot <- wt_plot[which(wt_plot$external_gene_name != "Adora2a"), ]
wt_plot <- wt_long2[which(wt_long2$autophagy==TRUE), ]

p <- ggplot(wt_plot, aes(x=external_gene_name, y=norm_reads, color=group)) +
  geom_violin() +
  geom_dotplot(binaxis="y", stackdir="center") +
  facet_grid(cols=vars(group), scales="free", space='free') +
  theme_classic() +
  theme(axis.text.x = element_text(angle = 90))
p



# heatmap for asyn
asyn_plot <- asyn_long2[which(asyn_long2$bdnf==TRUE), ]

asyn_plot <- asyn_long2[which(asyn_long2$microglia_activation==TRUE), ]
```

```
asyn_plot <- asyn_long2[which(asyn_long2$astroglia_activation==TRUE), ]
asyn_plot <- asyn_plot[which(asyn_plot$external_gene_name != "Adora2a"), ]

asyn_plot <- asyn_long2[which(asyn_long2$autophagy==TRUE), ]

asyn_matrix <- subset(asyn_plot, select=c("external_gene_name", "track_ID",
"norm_reads", "group"))
asyn_matrix$track_ID <- as.character(asyn_matrix$track_ID)
asyn_matrix <- unite(asyn_matrix, col=group_ID, c("group", "track_ID"), sep="-")

p <- ggplot(asyn_matrix, aes(x=group_ID, y=external_gene_name, fill=norm_reads)) +
  geom_tile(color = "black") +
  scale_fill_gradient2(low = "#075AFF",
                       mid = "#FFFFCC",
                       high = "#FF0000") +
#  coord_fixed() +
  theme_classic() +
  theme(axis.text.x = element_text(angle = 90))
p
```
