# Supplementary material for: Intermittent fasting reduces alpha-synuclein pathology and functional decline in a mouse model of Parkinson’s disease
Source: Nat Commun. 2025 May 14;16:4470. doi: 10.1038/s41467-025-59249-5 (PMC12078643; doi:10.1038/s41467-025-59249-5)
Supplement: Supplementary file 2 — Reporting Summary [file 41467_2025_59249_MOESM2_ESM.pdf]

nature portfolio

Corresponding author(s): Björn Falkenburger

Last updated by author(s): Apr 1, 2025

Reporting Summary

Nature Portfolio wishes to improve the reproducibility of the work that we publish. This form provides structure for consistency and transparency in reporting. For further information on Nature Portfolio policies, see our Editorial Policies and the Editorial Policy Checklist.

Statistics

For all statistical analyses, confirm that the following items are present in the figure legend, table legend, main text, or Methods section.

n/a	Confirmed	
☐	☒	The exact sample size (*n*) for each experimental group/condition, given as a discrete number and unit of measurement
☐	☒	A statement on whether measurements were taken from distinct samples or whether the same sample was measured repeatedly
☐	☒	The statistical test(s) used AND whether they are one- or two-sided<br>*Only common tests should be described solely by name; describe more complex techniques in the Methods section.*
☐	☒	A description of all covariates tested
☐	☒	A description of any assumptions or corrections, such as tests of normality and adjustment for multiple comparisons
☐	☒	A full description of the statistical parameters including central tendency (e.g. means) or other basic estimates (e.g. regression coefficient) AND variation (e.g. standard deviation) or associated estimates of uncertainty (e.g. confidence intervals)
☐	☒	For null hypothesis testing, the test statistic (e.g. *F*, *t*, *r*) with confidence intervals, effect sizes, degrees of freedom and *P* value noted<br>*Give P values as exact values whenever suitable.*
☒	☐	For Bayesian analysis, information on the choice of priors and Markov chain Monte Carlo settings
☐	☒	For hierarchical and complex designs, identification of the appropriate level for tests and full reporting of outcomes
☒	☐	Estimates of effect sizes (e.g. Cohen's *d*, Pearson's *r*), indicating how they were calculated

Our web collection on statistics for biologists contains articles on many of the points above.

Software and code

Policy information about availability of computer code

Data collection	No specific softwares were used to collect data.
Data analysis	All codes, softwares and data analysis method are defined in the manuscript. Code used for transcriptomic analysis is included as supplement.

For manuscripts utilizing custom algorithms or software that are central to the research but not yet described in published literature, software must be made available to editors and reviewers. We strongly encourage code deposition in a community repository (e.g. GitHub). See the Nature Portfolio guidelines for submitting code & software for further information.

Data

Policy information about availability of data

All manuscripts must include a data availability statement. This statement should provide the following information, where applicable:

- Accession codes, unique identifiers, or web links for publicly available datasets
- A description of any restrictions on data availability
- For clinical datasets or third party data, please ensure that the statement adheres to our policy

Transcriptomic data is on GEO: https://www.ncbi.nlm.nih.gov/geo/query/acc.cgi?acc=GSE252874
All other data is included in the manuscript or available from the corresponding author.

Research involving human participants, their data, or biological material

Policy information about studies with human participants or human data. See also policy information about sex, gender (identity/presentation), and sexual orientation and race, ethnicity and racism.

Reporting on sex and gender	Sex of animals are defined in the method section, both males and females were used.
Reporting on race, ethnicity, or other socially relevant groupings	No human data is included.
Population characteristics	n.a.
Recruitment	n.a.
Ethics oversight	Bioethical Committee of Landesdirektion Sachsen, Germany

Note that full information on the approval of the study protocol must also be provided in the manuscript.

Field-specific reporting

Please select the one below that is the best fit for your research. If you are not sure, read the appropriate sections before making your selection.

[x] Life sciences [ ] Behavioural & social sciences [ ] Ecological, evolutionary & environmental sciences

For a reference copy of the document with all sections, see nature.com/documents/nr-reporting-summary-flat.pdf

Life sciences study design

All studies must disclose on these points even when the disclosure is negative.

Sample size	Sample size was determined by G-power analysis.
Data exclusions	One animal was excluded from the transcriptomics analysis due to lack of transgene expression.
Replication	For histological analysis, 4-7 animal per group were used. For biochemical analysis, 5 animals per group were used. For transcriptomics analysis, 3 animals per group were used (one rAAV2/7-GFP animal was excluded due to lack of transgene expression). Experiments were not replcated on the exact same settings (3R rule for animal experimentation), however, the same experimental paradigm was applied on different cohorts (different age, genotype or collected for different analysis methods).
Randomization	1) Animals were randomly allocated to cages. 2) Animals were randomly allocated to experimental groups. 3) Brains received a four digit random code, that was resolved only at the final analysis.
Blinding	During data collections, researchers were blinded to experimental grouping by using a four digit random code. Blinding was resolved for the final analysis.

Reporting for specific materials, systems and methods

We require information from authors about some types of materials, experimental systems and methods used in many studies. Here, indicate whether each material, system or method listed is relevant to your study. If you are not sure if a list item applies to your research, read the appropriate section before selecting a response.

Materials & experimental systems

n/a	Involved in the study	
[ ]	[x]	Antibodies
[x]	[ ]	Eukaryotic cell lines
[x]	[ ]	Palaeontology and archaeology
[ ]	[x]	Animals and other organisms
[x]	[ ]	Clinical data
[x]	[ ]	Dual use research of concern
[x]	[ ]	Plants

Methods

n/a	Involved in the study	
[x]	[ ]	ChIP-seq
[x]	[ ]	Flow cytometry
[x]	[ ]	MRI-based neuroimaging

Antibodies

Antibodies used	Antibodies and cataloge numbers are included in SI Table.

Validation	Antibodies were not specifically validated. Primary and secondary antibody controls were included, where lack of antibody resulted in no staining.

Animals and other research organisms

Policy information about studies involving animals; ARRIVE guidelines recommended for reporting animal research, and Sex and Gender in Research

Laboratory animals	Details are descibed in the method section.
Wild animals	n.a.
Reporting on sex	Both males and females were used in this study.
Field-collected samples	This study did not involve samples collected from the field.
Ethics oversight	The animal experiments were approved by the Bioethical Committee of Landesdirektion Sachsen, Germany.

Note that full information on the approval of the study protocol must also be provided in the manuscript.

Plants

Seed stocks	n.a.
Novel plant genotypes	n.a.
Authentication	n.a.
